# Universal Patterns of Selection in Cancer and Somatic Tissues

**DOI:** 10.1016/j.cell.2017.09.042

**Published:** 2017-11-16

**Authors:** Iñigo Martincorena, Keiran M. Raine, Moritz Gerstung, Kevin J. Dawson, Kerstin Haase, Peter Van Loo, Helen Davies, Michael R. Stratton, Peter J. Campbell

**Affiliations:** 1 Wellcome Trust Sanger Institute, Hinxton CB10 1SA, Cambridgeshire, UK; 2 European Molecular Biology Laboratory, European Bioinformatics Institute EMBL-EBI, Hinxton CB10 1SD, UK; 3 The Francis Crick Institute, London NW1 1AT, UK; 4 Department of Human Genetics, University of Leuven, Leuven 3000, Belgium; 5 Department of Haematology, University of Cambridge, Cambridge CB2 2XY, UK

**Keywords:** cancer, genomics, evolution, mutations, selection

## Abstract

Cancer develops as a result of somatic mutation and clonal selection, but quantitative measures of selection in cancer evolution are lacking. We adapted methods from molecular evolution and applied them to 7,664 tumors across 29 cancer types. Unlike species evolution, positive selection outweighs negative selection during cancer development. On average, <1 coding base substitution/tumor is lost through negative selection, with purifying selection almost absent outside homozygous loss of essential genes. This allows exome-wide enumeration of all driver coding mutations, including outside known cancer genes. On average, tumors carry ∼4 coding substitutions under positive selection, ranging from <1/tumor in thyroid and testicular cancers to >10/tumor in endometrial and colorectal cancers. Half of driver substitutions occur in yet-to-be-discovered cancer genes. With increasing mutation burden, numbers of driver mutations increase, but not linearly. We systematically catalog cancer genes and show that genes vary extensively in what proportion of mutations are drivers versus passengers.

## Highlights

• Unlike the germline, somatic cells evolve predominantly by positive selection
• Nearly all (∼99%) coding mutations are tolerated and escape negative selection
• Exome-wide estimates of the total number of driver coding mutations per tumor
• Half of the coding driver mutations occur outside of known cancer genes

## Graphical Abstract

**Figure:**
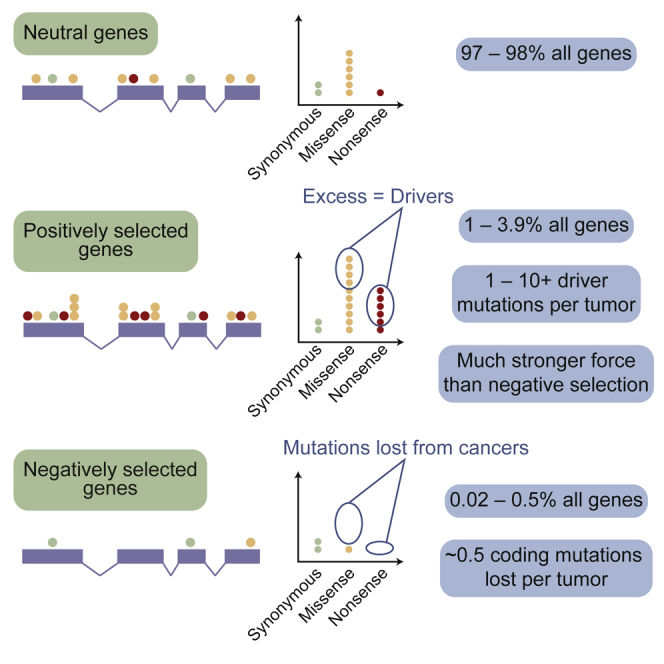

## Teaser

Adapting an evolutionary genomics approach to cancer highlights a limited impact of negative selection on cancer genomes and significant variations in the proportion of coding driver mutations per tumor among different tumor types.

## Introduction

Somatic cells accumulate mutations throughout life. These mutations can be classified into those that confer a selective advantage on the cell, increasing survival or proliferation (so-called “driver” mutations), those that are selectively neutral, and those that are disadvantageous to the cell and result in its death or senescence. Cancer is one end-product of somatic evolution, in which a single clonal lineage acquires a complement of driver mutations that enables the cells to evade normal constraints on cell proliferation, invade tissues, and spread to other organs.

While the general principles of cancer evolution have been well documented for some decades (Cairns, 1975, Nowell, 1976), fundamental questions remain unanswered. We still do not have accurate estimates of the number of mutations required to drive a cancer and whether this varies extensively across tumor types or with different mutation rates (Martincorena and Campbell, 2015). One approach to this question has been to use age-incidence curves to estimate the number of rate-limiting steps required for a cancer to develop (Armitage and Doll, 1954, Tomasetti et al., 2015), with the implicit assumption of a one-to-one correspondence between rate-limiting steps and driver mutations. However, not all driver mutations need be rate-limiting (Yates et al., 2015), nor need every rate-limiting event be a driver mutation (Martincorena and Campbell, 2015). A second approach to estimating the number of driver mutations has simply been to count the mutations occurring in known cancer genes, but this is limited by incomplete lists of cancer driver genes and by the presence of passenger mutations in cancer genes. Thus, despite its fundamental importance and the sequencing of thousands of cancer genomes, the question of how many somatic mutations drive a cancer remains unresolved.

A second major gap in our understanding of cancer evolution is that we have not yet been able to measure the importance of negative selection in shaping the cancer genome and to what extent somatic lineages expire due to the effects of deleterious mutations. Detection of negative selection in cancer genomes is an important endeavor as it may help identify genes essential for cancer growth and patterns of synthetic lethality, potentially yielding new therapeutic targets. With increasing interest in the role of neoantigens created by somatic mutations shaping the immune response to cancer (McGranahan et al., 2016, Rajasagi et al., 2014, Rooney et al., 2015), we might expect that purifying selection would suppress clones with mutations that elicit a strong immune reaction.

While we have increasingly detailed lists of cancer genes (Kandoth et al., 2013, Lawrence et al., 2014, Vogelstein et al., 2013), it is not always straightforward to identify which mutations in those genes are true driver mutations nor how many mutations in other genes might be drivers. This will become an increasingly important question as cancer genome sequencing moves into routine clinical practice—therapeutic decision support for an individual patient critically depends on accurate identification of which specific mutations drive that person’s cancer (Gerstung et al., 2017).

In this study, we address these open questions by adapting methods from molecular evolution to the study of cancer genomes. The key advance in the models we develop is that we can directly enumerate the excess or deficit of mutations in a given gene, a group of genes, or even the whole exome, compared to the expectation for the background mutational processes. This enables us to provide robust estimates of the total number of coding driver mutations across cancers, how many coding point mutations are lost through negative selection, and a detailed dissection of the distribution of driver mutations in individual cancer genes across different tumor types.

## Results

### Quantitative Assessment of Positive and Negative Selection

Detection of selection in traditional comparative genomics typically requires a measure of the expected density of selectively neutral mutations in a gene. In the context of cancer, a gene under positive selection will carry an extra complement of driver mutations in addition to neutral (passenger) mutations—it is this recurrence of mutations across cancer patients that has underpinned discoveries of cancer genes from the Philadelphia chromosome to modern genomic studies (Martincorena and Campbell, 2015). A gene subject to purifying selection of deleterious mutations would have fewer mutations than expected under neutrality (Greenman et al., 2006).

Building on previous work (Greenman et al., 2006, Martincorena et al., 2015, Yang et al., 2003), we use dN/dS, the normalized ratio of non-synonymous to synonymous mutations, to quantify selection in cancer genomes. This relies on the assumption that the vast majority of synonymous mutations are selectively neutral and hence a good proxy to model the expected mutation density (we address the accuracy of this assumption later; see also STAR Methods). dN/dS has a long history in the study of selection in species evolution (Goldman and Yang, 1994, Nei and Gojobori, 1986, Yang and Bielawski, 2000), but several modifications are required for somatic evolution.

**Figure S1:**
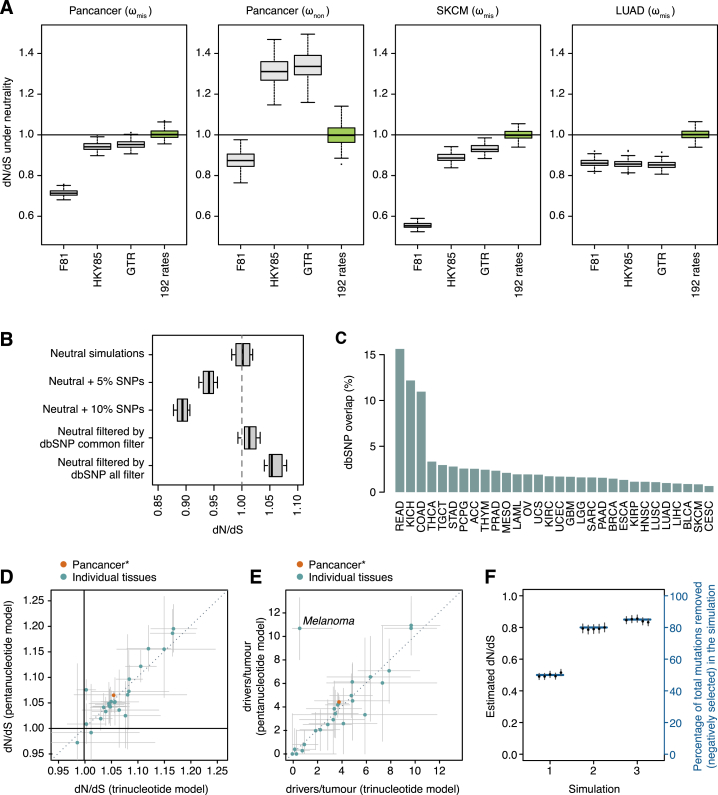
Impact of Different Confounding Factors on Analyses of Selection, Related to Figures 1–5 This includes simplistic substitution models, SNP contamination, SNP filtering and inadequate background models of the variation of the mutation rate. (A) Impact of simplistic mutation models on the accuracy of dN/dS in different scenarios. Each boxplot represents the dN/dS ratios estimated from 100 neutral simulations of 10,000 random coding substitutions. To exemplify the impact on dN/dS of different mutational spectra, we simulated neutral datasets using the trinucleotide spectra observed in the three different cohorts of samples (pancancer, melanoma and lung adenocarcinoma). Different panels depict dN/dS ratios for missense (ω_mis_) or nonsense (ω_non_) mutations. (B) Simulations of the impact on dN/dS of germline SNP contamination and SNP over-filtering in catalogs of somatic mutations. 10 neutral datasets were generated by local randomization of 607 cancer whole-genomes (Alexandrov et al., 2013). Datasets with varying degrees of germline SNP contamination were simulated by adding 5% or 10% of germline common SNPs (minor allele frequency > = 5%) from 1000 genomes phase 3 (Auton et al., 2015) to the neutral simulations. Datasets with varying levels of SNP over-filtering were simulated by removing any mutation from the neutral datasets that overlapped a polymorphic site in dbSNP build 146 (either using common sites or all sites) (Sherry et al., 2001). (C) Percentage of mutations from the public TCGA catalogs of somatic calls that overlap a common dbSNP site. Based on simulations, an overlap of 1%–3% might be expected depending on the dominant mutational signatures present in a dataset, but several public TCGA catalogs show a much higher overlap suggesting extensive germline SNP contamination. As predicted from (B), this leads to an artifactual signal of negative selection in these datasets (STAR Methods). (D) Consistency between genome-wide dN/dS estimates using the trinucleotide and pentanucleotide substitution models across cancer types. Green dots represent genome-wide dN/dS estimates for each cancer type separately, and the orange dot depicts the pancancer estimates (using the 24 cancer types with *CaVEMan* mutation calls). (E) Corresponding estimates of the average number of driver coding substitutions per tumor. For the purpose of estimating the excess of mutations from dN/dS ratios, dN/dS values below 1 are set to 1. Error bars depict 95% CIs. (F) Simulations demonstrating the validity of estimating dN/dS at a cohort level, in heterogeneous cohorts of samples without patient-specific substitution models. The three scenarios simulated include extreme examples of heterogeneous mixtures of samples with variable signatures, numbers of mutations and selection. In each scenario, the correct fraction of mutations removed by negative selection across samples is shown as a blue horizontal line (right y axis). Estimated dN/dS values from five simulations of each scenario are shown as dots with CIs (left y axis).

**Figure S2:**
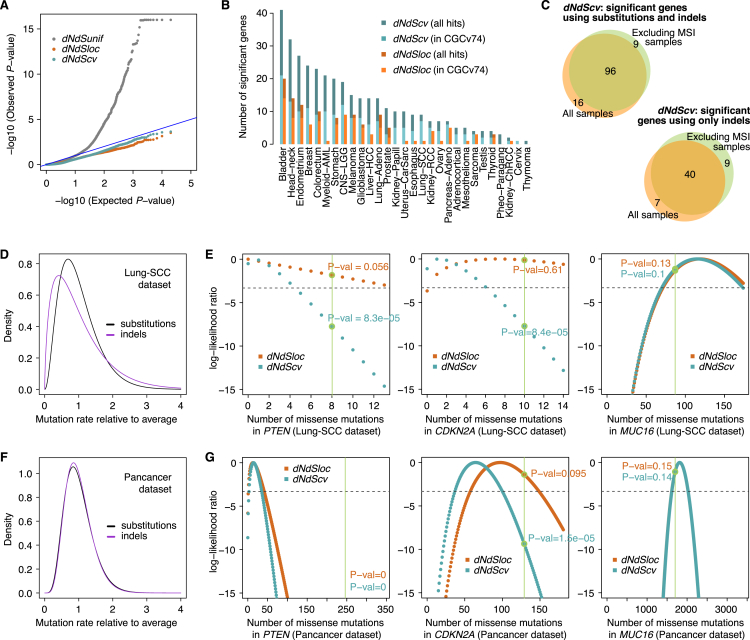
Evaluation of the Relative Performance of the Three Different dN/dS Models for the Detection of Positive Selection at Gene Level, Related to Figure 2 (A) QQ-plots for the different dN/dS models on a neutral dataset obtained by randomization of 107 melanoma whole-genomes from ICGC (STAR Methods). The *dNdSunif* model shows a great inflation of low *P*-values, leading to a large number of false positives after multiple testing correction (368 genes with q-value < 0.05), and should be generally avoided. In contrast, both *dNdSloc* and *dNdScv* behave as expected for a neutral dataset, yielding no significant hits after multiple testing correction. (B) Sensitivity of *dNdScv* and *dNdSloc*. The bar plot depicts the number of significant genes (q-value < 0.05) identified by both methods in the 29 TCGA datasets. Bars colored in a lighter shade show the number of significant genes that are present in the Cancer Gene Census version 73 (Forbes et al., 2015). *dNdScv* shows good specificity and sensitivity under all tested conditions (STAR Methods). (C) Comparison of the number of significant genes found by *dNdScv* (top) and the indel model (bottom) in their default configuration (*unique-sites* model for indels) when including and excluding MSI samples. (D–G) Gamma distributions and log-likelihood surfaces of *dNdScv* on a number of genes and datasets. (D,F) Density functions of the Gamma distributions for substitutions and indels inferred by the negative binomial regression in *dNdScv* for two datasets (Lung-SCC and Pancancer). The Gamma distributions shown have a mean = 1, showing the spread around the mean observed across genes in each dataset. This reflects the extent of the variation of the mutation rate across genes that remains unexplained by sequence composition, signatures and covariates. (E,G) Log-likelihood ratio values for the number of missense mutations in three genes (*PTEN*, *CDKN2A* and *MUC16*) in the Lung-SCC (n = 167 samples) and Pancancer datasets (n = 7,664) under *dNdSloc* and *dNdScv*. The real observed number of missense mutations in each gene and dataset is shown as a vertical green line. The figures show how in small genes and/or small datasets, *dNdScv* has much narrower curves and much more significant *P*-values for cancer genes thanks to the Gamma constraint, while *dNdScv* and *dNdSloc* converge when the local number of synonymous mutations is sufficiently high. This adaptive behavior of *dNdScv* results from the joint likelihood equation.

The first critical refinement is more comprehensive models for context-dependent mutational processes (Alexandrov et al., 2013, Greenman et al., 2006, Yang et al., 2003). Traditional implementations of dN/dS use simplistic mutation models that lead to systematic bias in dN/dS ratios and can cause incorrect inference of positive and negative selection (Figure S1)—such biases have affected previous studies in this area (Ostrow et al., 2014). Therefore, we use a model with 192 rate parameters that accounts for all 6 types of base substitution, all 16 combinations of the bases immediately 5′ and 3′ to the mutated base, and transcribed versus non-transcribed strands of the gene (Figure S1A). A second refinement is the addition of other types of non-synonymous mutations beyond missense mutations, including nonsense and essential splice site mutations (Greenman et al., 2006), and a method for small insertions and deletions (indels). Third, extreme caution was exercised during variant calling to avoid biases emerging from germline variants, because these have a much lower dN/dS ratio than somatic mutations. Misannotation of a germline polymorphism as a somatic mutation will bias somatic dN/dS downward; excessively filtering true somatic mutations that occur at positions known to be polymorphic in the population will bias somatic dN/dS upward (Figure S1B). For example, we have seen that germline contamination of the public mutation catalogs from several datasets in The Cancer Genome Atlas [TCGA], such as colorectal cancer and chromophobe renal cell carcinoma, generates a false signal of negative selection (Figure S1C). Fourth, to detect selection at the level of individual genes reliably, and particularly for driver gene discovery, we refined dN/dS to consider the variation of the mutation rate along the human genome. A simple way to do so is estimating a separate mutation rate for every gene (Wong et al., 2014), but this approach has low sensitivity with typical sample sizes. Instead, we developed a statistical model (*dNdScv*) that combines the local observed synonymous mutation rate with a regression model using covariates that predict the variable mutation rate across the genome (Lawrence et al., 2013, Polak et al., 2015, Schuster-Böckler and Lehner, 2012). This approach has the advantage of optimizing the balance between local and global data on estimating background mutation rates to provide a statistically efficient inference framework for departures from neutrality (Figure S2).

In order to study the landscape of positive and negative selection in cancer, we applied these approaches to a collection of 7,664 tumors from 29 cancer types from TCGA (Table S1). Somatic mutations were re-called with our in-house algorithms across 24 cancer types to ensure comparability across tumor types and avoid biases from germline polymorphisms.

### A Universal and Distinct Pattern of Selection in Cancer

**Figure 1:**
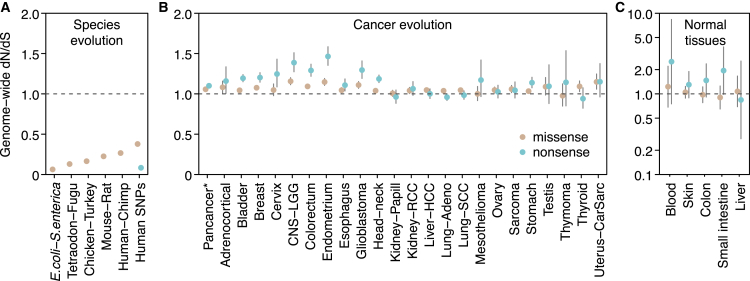
Genome-wide dN/dS Ratios Show a Distinct Pattern of Selection Universally Shared across Cancer Types (A) Species evolution: median dN/dS ratios across genes for missense mutations (data from Martincorena et al. [2012] and Ensembl). Data on germline human SNPs are from the 1,000 genomes phase 3 (Auton et al., 2015), restricted to SNPs with minor allele frequency ≥5%. (B) Cancer evolution: genome-wide dN/dS values for missense and nonsense mutations across 23 cancer types. (C) Somatic mutations in normal tissues (data from Blokzijl et al., 2016, Martincorena et al., 2015, Welch et al., 2012). Error bars depict 95% CIs. See also Figure S1 and Table S1.

Comparative genomic studies of related species typically reveal very low dN/dS ratios, reflecting that the majority of germline non-synonymous mutations are removed by negative selection over the course of evolution (Ostrow et al., 2014). For example, comparison of orthologous genes from *Escherichia coli* and *Salmonella enterica* yields an average dN/dS∼0.06 across genes. This indicates that at least ∼94% of missense mutations have been removed by negative selection. The dN/dS ratio for nonsense mutations in common human germline polymorphisms is similarly low (dN/dS∼0.08). dN/dS ratios vary across species but a pattern of overwhelming negative selection invariably characterizes species evolution (Figure 1A).

In stark contrast, cancer evolution shows a pattern in which dN/dS ratios are close to, but slightly above, 1 (Figure 1B). This pattern is universally shared across tumor types studied here and applies to both missense and truncating substitutions (nonsense and essential splice site mutations). This indicates that mutations under positive selective pressure are somewhat more numerous in cancers than mutations under negative selection, but the overall picture is close to neutrality. Importantly, similar values of dN/dS around or above 1 are found in somatic mutations detected in healthy tissues, including blood, skin, liver, colon, and small intestine (Blokzijl et al., 2016, Martincorena et al., 2015, Welch et al., 2012) (Figure 1C). Although these data are still limited, dN/dS∼1 appears to characterize somatic evolution in normal somatic tissues as well as all cancers that we have studied so far.

### Identification of Genes under Positive Selection

**Figure 2:**
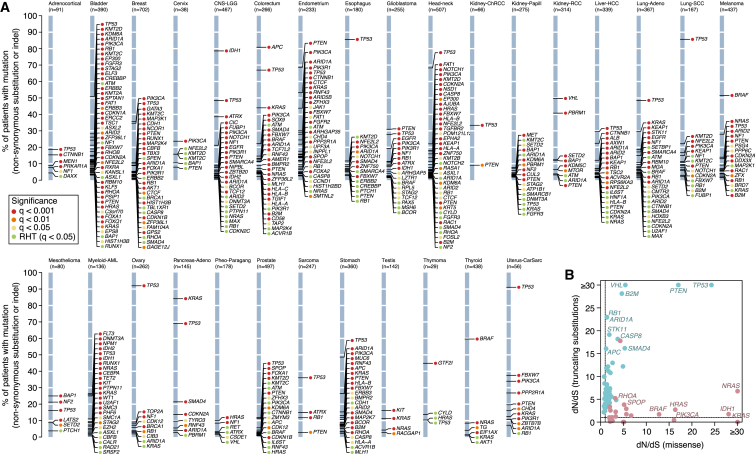
Positively Selected Genes (Drivers) in Cancer Genomes (A) List of genes detected under significant positive selection (dN/dS >1) in each of the 29 cancer types. Y axes show the percentage of patients carrying a non-synonymous substitution or an indel in each gene. The color of the dot reflects the significance of each gene. RHT, restricted hypothesis testing on known cancer genes (Table S2). (B) Pancancer dN/dS values for missense and nonsense mutations for genes with significant positive selection on missense mutations (depicted in red) and/or truncating substitutions. See also Figures S1 and S2.

By definition, cancer genes are genes under positive selection in tumor cells. To show the ability of dN/dS to uncover cancer genes, we used *dNdScv* to identify genes for which dN/dS was significantly higher than 1, both across all 7,664 cancers and for each tumor type individually (Figure 2A). This revealed 179 cancer genes under positive selection at 5% false discovery rate. Of these, 54% are canonical cancer genes present in the Cancer Gene Census (Forbes et al., 2015). Using restricted hypothesis testing (Lawrence et al., 2014) on a priori known cancer genes identifies an additional 24 driver genes. Evaluation of genes not present in the Census reveals that most have been previously reported as cancer genes, have been found in other pan-cancer analyses, or have clear links to cancer biology (Kandoth et al., 2013, Lawrence et al., 2014, Rubio-Perez et al., 2015) (Table S2). Novel candidate cancer genes include *ZFP36L1* and *ZFP36L2*, which have recently been shown to promote cellular quiescence and suppress S-phase transition during B cell development (Galloway et al., 2016). We find higher than expected rates of inactivating mutations in the two genes in several tumor types, suggesting that they have a tumor suppressor role. Other novel tumor suppressor genes identified here include *KANSL1*, a scaffold protein for histone acetylation complexes (Dias et al., 2014), *BMPR2*, a receptor serine/threonine kinase for bone morphogenetic proteins, *MAP2K7*, involved in MAP-kinase signaling, and *NIPBL*, a member of the cohesin complex.

As expected, depending on whether nonsense or missense mutations predominate, genes generally fall into two classes: oncogenes, with strong selection on missense mutations, or tumor suppressor genes, with stronger selection on truncating mutations (Figure 2B). Significant dN/dS ratios reach very high values in frequently mutated driver genes, often higher than 10 or even 100 (Figure 2B). This gives quantitative information about the proportion of driver mutations. For example, dN/dS = 10 for a gene evidences that there are ten times more non-synonymous mutations in the gene than expected under neutral accumulation of mutations, indicating that at least ∼90% of the non-synonymous mutations in the gene are genuine driver mutations (Greenman et al., 2006).

### Negative Selection Is Largely Absent for Coding Substitutions

While some somatic mutations can confer a growth advantage, others may impair cell survival or proliferation. Clones carrying such mutations would senesce or die, with the result that the mutation would be lost from the catalog of variants seen in the eventual cancer. This negative or purifying selection will lead to dN/dS <1 in a given gene or set of genes if it occurs at appreciable rates. Negative selection on somatic mutations has been long anticipated (Beckman and Loeb, 2005, McFarland et al., 2014, Nowell, 1976) but not yet reliably documented in cancer genomes. This is due to the fact that statistical detection of lower mutation density than expected by chance requires large datasets and very careful consideration of mutation biases and germline SNP contamination.

**Figure 3:**
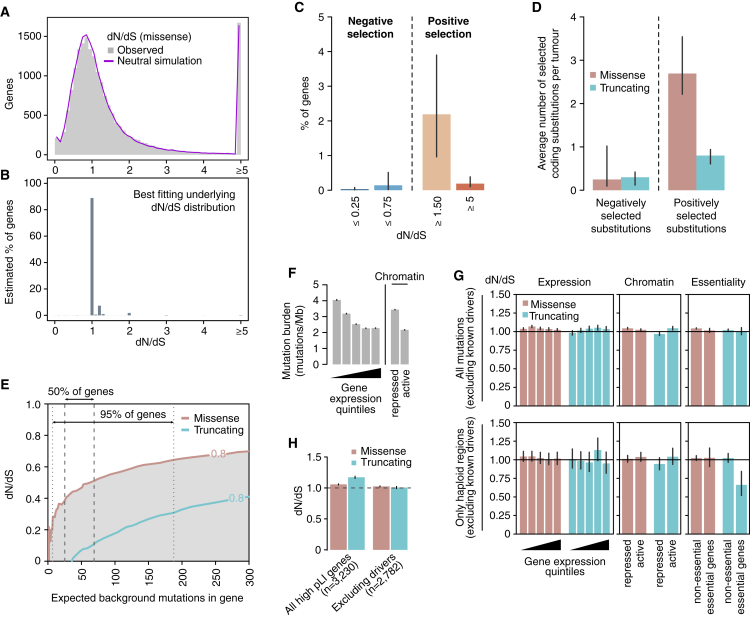
Negative Selection in Cancer (A) Distributions of dN/dS values per gene for missense mutations in non-LOH regions. The real distribution is shown in gray and the distribution observed in a neutral simulation is shown in purple. (B) Underlying distribution of dN/dS values across genes inferred from the observed distribution. (C) Estimated percentage of genes under different levels of positive and negative selection based on the inferred dN/dS distribution in (B). (D) Average number of selected mutations per tumor based on the inferred distributions of dN/dS across genes, combining missense and truncating mutations from all copy number regions. Error bars depict 95% CIs. (E) Power calculation for the statistical detection of negative selection (dN/dS <1) as a function of the extent of selection (dN/dS) and the neutrally-expected number of mutations in a gene in a cohort. Shaded areas under the curves reflect power >80%. Vertical lines indicate the range in which the middle 50% and 95% of genes are in the dataset of 7,664 tumors. (F) Average mutation burden in genes grouped according to gene expression quintile and chromatin state. (G) Average dN/dS values for genes grouped according to gene expression quintile, chromatin state, and essentiality. (H) Average dN/dS values for all mutations in genes found to be haploinsufficient in the human germline, including and excluding putative driver genes. Haploinsufficient genes are defined as those having a pLI score >0.9 in the ExAC database (Lek et al., 2016). See also Figures S1 and S3.

**Figure S3:**
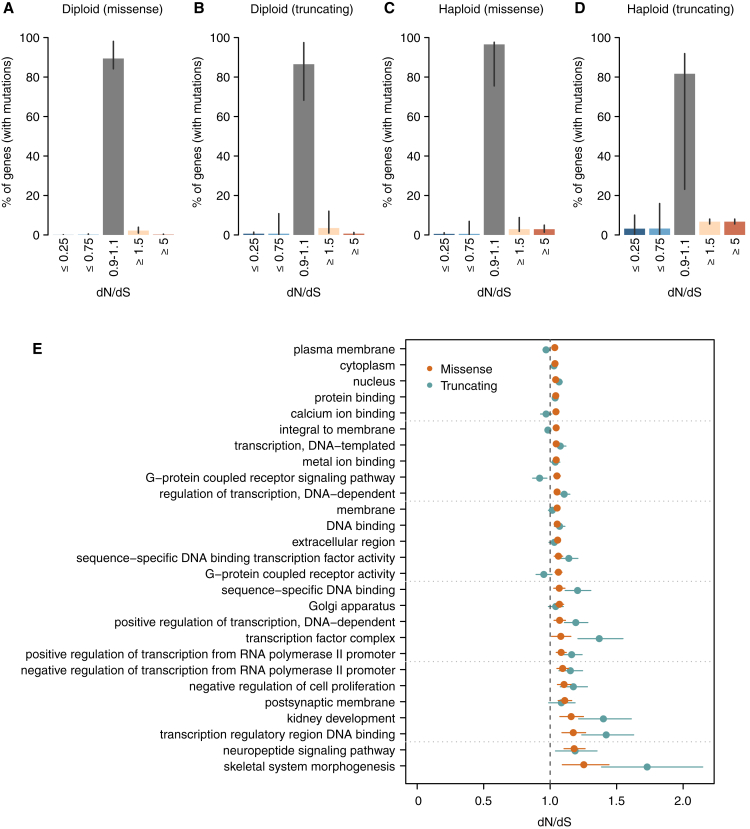
Supplementary Analyses on Negative Selection, Related to Figure 3 (A–D) dN/dS distributions inferred for different mutation types and copy number states. These distributions, obtained as described for Figure 3C, represent the percentage of genes estimated to be under a certain selection regime. The four distributions correspond to: missense (A) and truncating (B) substitutions in regions without loss of heterozygosity, and missense and truncating substitutions in haploid regions (C and D, respectively). Note that (A) is an extension of Figure 3C, with an added middle bar for genes with dN/dS very close to 1 (0.9-1.1), which can be considered to evolve largely neutrally. Only samples with *CaVEMan* mutation calls, excluding melanoma samples, were considered for this analysis for the reasons explained in the Methods. For each figure, all mutations with the appropriate ploidy were included in the analysis and only genes with at least one mutation (either synonymous or non-synonymous) participate in the fitting of dN/dS distributions. Hence, the percentages of genes shown in the y-axes are relative to the total number of genes with at least one mutation in regions with the ploidy considered in each figure. Error bars depict 95% CIs. (E) Gene ontology groups deviating significantly from neutrality after removing known cancer genes. 27 gene ontology classes are found to be under significant positive selection after comprehensively removing 987 known putative cancer genes. This suggests the presence of undiscovered cancer genes in these functional groups. No gene ontology class was found to be under significant negative selection. Error bars depict 95% CIs.

To determine the potential extent of negative selection, we first studied the distribution of observed dN/dS values per gene. There is considerable spread of these observed values around the neutral peak at dN/dS = 1.0 (Figure 3A), which at face value might suggest that many genes are under positive or negative selection. However, the limited numbers of mutations per gene make individual dN/dS values noisy, and we find that the observed distribution almost exactly matches that seen in simulations under a model where all genes are neutral. To formally estimate the fraction of genes under negative selection, we infer the underlying distribution of dN/dS values from the observed data using a binomial mixture model (Figures 3B and 3C). We find that the vast majority of genes are expected to accumulate point mutations near neutrally, with dN/dS∼1. A small fraction of genes (∼2.2%; confidence interval (CI)_95%_ = 1.0%–3.9%) show dN/dS ≥1.5, consistent with current estimates of the numbers of cancer genes. Only a tiny fraction of genes (∼0.14%; CI_95%_ = 0.02%–0.51%), equating to a few tens of genes, are estimated to exhibit negative selection with dN/dS ≤0.75 (Figures 3C and S3A–S3D).

These distributions also enable us to obtain approximate estimates of the average number of coding substitutions lost by negative selection per tumor (Figure 3D). On average, across this diverse collection of tumors, less than one coding substitution per tumor (0.55/patient; CI_95%_ = 0.31–1.16) has been lost by negative selection, accounting for <1% of all coding mutations. We note the formal possibility that dN/dS = 1 can occur when the numbers of positively and negatively selected mutations in a given gene are exactly balanced. This could lead us to underestimate the extent of negative selection but only if a large number of genes showed such an exact balance, which seems unlikely.

Although negative selection in cancers might be weak globally, it remains possible that negative selection may act in very specific scenarios, genes, or gene sets. No single gene had a dN/dS significantly <1 after multiple hypothesis testing correction, even if we boost our power by performing restricted hypothesis testing on 1,734 genes identified by in vitro screens as essential (Blomen et al., 2015). To address the possibility of making a type II inference error, we evaluated our statistical power to detect negative selection at the level of individual genes in this dataset (Figure 3E). We found that there is enough power to detect negative selection at dN/dS <0.5 on missense mutations for most genes in the genome, but we have less power for detecting negative selection acting on truncating mutations (Figure 3E). Thus, the lack of significant negative selection in any gene in the current dataset reveals that negative selection would be weaker than these detection limits.

We next examined whether specific groups of genes might be subject to negative selection, after excluding 987 putative cancer genes to avoid obscuring the signal of negative selection. Sets of genes that may be expected to be under stronger negative selection include highly expressed genes or genes in active chromatin regions. Lower mutation density has been observed in cancer genomes in highly expressed genes and open chromatin (Figure 3F) (Pleasance et al., 2010b, Schuster-Böckler and Lehner, 2012) and some have suggested that this may be a signal of negative selection (Lee et al., 2010). However, we found that dN/dS values are virtually indistinguishable from neutrality for both missense and truncating substitutions across gene expression levels and chromatin states. This confirms that the lower density of mutations observed in open chromatin and highly expressed genes is due to lower mutation rates in these regions and not negative selection. The lack of detectable negative selection even extends to nonsense mutations in essential genes (Figure 3G; top panel). Gene sets grouped by gene ontology and functional annotation similarly revealed no clear evidence of negative selection (Figure S3E).

One reason for this unexpected weakness of negative selection in cancer could be that cancer cells typically carry two (or more) copies of most genes, reducing the impact of mutations inactivating a single gene copy. We used copy number data for the samples studied here to identify those coding mutations occurring in haploid regions of the genome. Strikingly, most missense and even truncating substitutions affecting the single remaining copy of a gene seem to accumulate at a near-neutral rate, suggesting that they are largely tolerated by cancer cells (Figure 3G; bottom panel). However, for essential genes in regions of copy number 1, nonsense substitutions do exhibit significantly reduced dN/dS, with approximately one-third of such variants lost through negative selection (dN/dS = 0.66, p value = 8.4 × 10^−4^, Figure 3G). This result is consistent with the recent observation of weak signals of purifying selection on hemizygous genomic regions (Van den Eynden et al., 2016).

Finally, analysis of mutations in human genes that are intolerant to heterozygous loss-of-function mutations in the germline also revealed no detectable negative selection in cancer cells. This applied similarly to both missense and truncating substitutions (Figure 3H).

Overall, these analyses show that negative selection in cancer genomes is much weaker than anticipated. With the exception of driver mutations, nearly all coding substitutions (∼99%) appear to accumulate neutrally during cancer evolution and are tolerated by cancer cells. Several factors are likely to contribute to the weakness of negative selection in cancer and somatic evolution, some highlighted before (McFarland et al., 2013). These include, among other factors: (1) the buffering effect of having two or more copies of most genes; (2) the fact that, for any given somatic lineage, a large number of genes are likely to be dispensable (Morley, 1995); (3) the frequent hitchhiking with driver mutations, which enables weakly deleterious mutations not yet expunged to be fixed in a cancer population; (4) moderately high mutation rates per division and asexual reproduction of cancer cells, which prevent deleterious mutations from being separable from other variants in the genome and lead to their progressive accumulation (known as Muller’s ratchet); and (5) differences in population size and structure, such as stem cell niches, which are likely to exacerbate genetic drift.

Immune surveillance is believed to be a relevant force shaping cancer evolution, potentially acting to purge clones carrying neoantigens generated by somatic mutations. Genomic studies have predicted that cancers typically carry tens of coding mutations that generate potential neoantigens (McGranahan et al., 2016, Rajasagi et al., 2014), with as many as 50% of non-synonymous mutations predicted to create a neoantigen (Rooney et al., 2015). The observation that ∼99% of somatic substitutions are tolerated and accumulate neutrally in cancer cells confirms that the vast majority of predicted neoantigens do not elicit an immune response capable of eradicating the clone in normal conditions, even if they could be exploited therapeutically (Strønen et al., 2016).

### Number of Driver Mutations per Tumor

**Figure 4:**
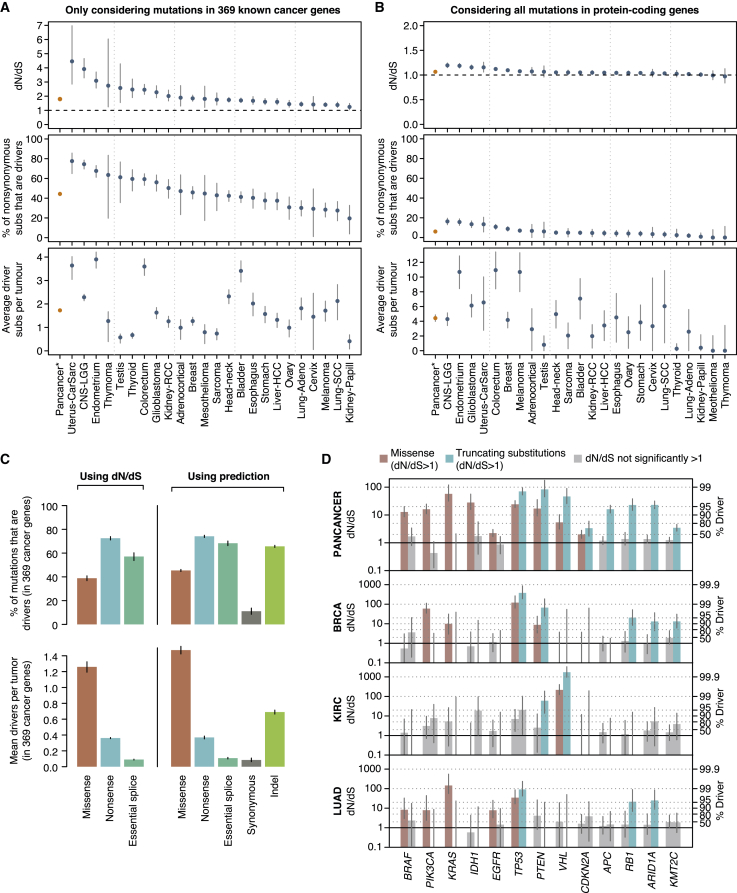
Average Number of Driver Mutations in Tumors with <500 Coding Mutations (A) Top: Global dN/dS values obtained for 369 known cancer genes (Table S3). This analysis uses a single dN/dS ratio for all non-synonymous substitutions (missense, nonsense, and essential splice site). Middle: Percentage of non-synonymous mutations that are drivers assuming negligible negative selection. Bottom: Average number of driver coding substitutions per tumor. Pancancer refers to the 24 cancer types with in-house mutation calls. (B) Same panels as (A) but including all genes in the genome. (A) and (B) were generated under the pentanucleotide substitution model for maximum accuracy. (C) Percentage (top) and mean absolute number (bottom) of driver mutations per tumor in 369 known cancer genes, using two different approaches: (1) dN/dS, and (2) fitting a Poisson regression model with covariates on putative passenger genes and using this to measure the excess of mutations in known cancer genes. This allows estimating the driver contribution of indels and synonymous mutations. (D) Left y axis: dN/dS values for missense and truncating substitutions for a series of driver genes and for different datasets. Right y axis: Corresponding estimates of the fraction of driver mutations. Grey bars depict dN/dS ratios not significantly different from one. Error bars depict 95% CIs. Generated using all samples with <3,000 coding mutations, as Figure 2. See also Figures S1 and S4.

**Figure 5:**
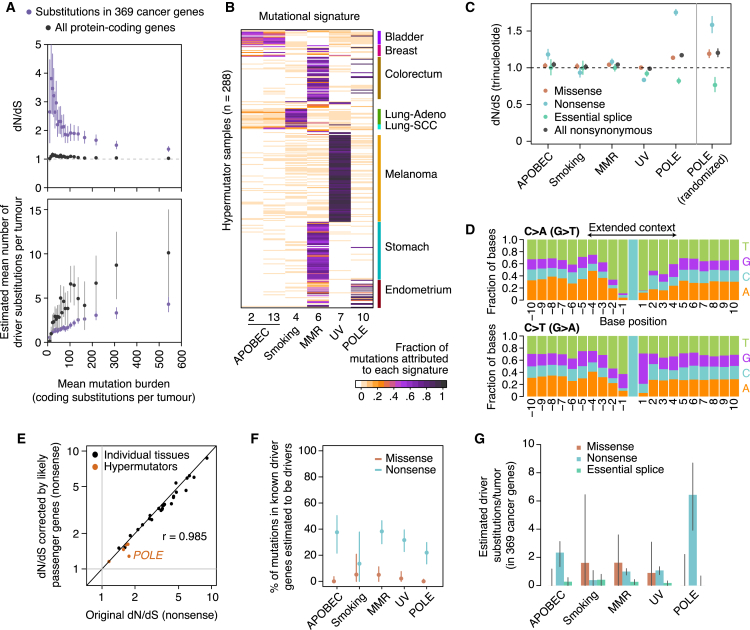
Selection in Hypermutator Tumors (A) dN/dS and estimated number of driver mutations per tumor grouping samples in 20 equal-sized bins according to mutation burden. This analysis excludes melanoma samples and uses a pentanucleotide substitution model to minimize mutational biases. (B) Heatmap depicting the fraction of mutations in 288 hypermutator samples (>1,000 mutations/exome) attributed to different mutational signatures (Alexandrov et al., 2013). (C) Left: dN/dS ratios (trinucleotide model) for each class of hypermutators. Right: dN/dS ratios from a neutral simulated dataset of *POLE* mutations. This neutral dataset was generated by randomizing all non-coding substitutions from five *POLE* hypermutator whole-genomes to a different site with an identical 9-nucleotide context, within 1-megabase of its original position. (D) Stacked bar plot showing the frequency of each base around C > A and C > T substitutions in *POLE* hypermutator tumors. (E–G) Conservative estimation of the fraction (F) and absolute number (G) of driver coding substitutions in known cancer genes. To obtain these estimates, dN/dS ratios for known cancer genes were normalized by those from putative passenger genes, to conservatively remove mutational biases from dN/dS. Application of this approach to our tissue-specific estimates in Figure 4A yields analogous results (E).

The number of driver mutations required to generate a tumor has been a long-standing question in cancer (Armitage and Doll, 1954, Martincorena and Campbell, 2015, Nordling, 1953, Tomasetti et al., 2015). The sequencing of thousands of cancer genomes has not clarified this question further because it remains unclear what fraction of non-synonymous mutations observed in known cancer genes are genuine driver mutations and how many driver mutations occur in cancer genes that are yet to be discovered. Given the weakness of negative selection, we can use dN/dS to estimate the average number of driver mutations per tumor. To obtain reliable estimates that are representative of the vast majority of tumors, we first restrict the analyses to non-hypermutator samples (defined here as samples with <500 coding mutations, accounting for 92% of all TCGA samples) (this section, Figures 4A–4C). We then describe additional analyses on hypermutator samples in a subsequent section (Figure 5).

**Figure S4:**
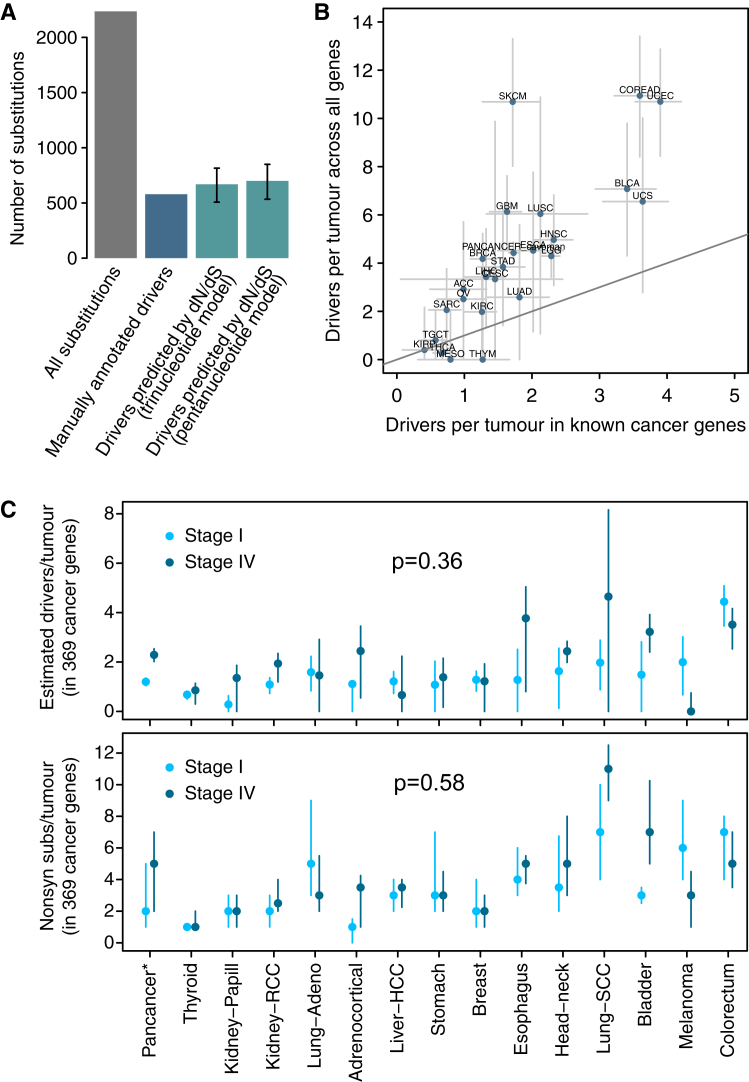
Supplementary Analyses on the Number of Coding Driver Substitutions per Tumor, Related to Figure 4 (A) Comparison of the number of coding driver substitutions estimated by dN/dS and the number estimated by manual annotation of driver mutations across 560 breast cancers. The figure depicts the total number of coding substitutions (gray bar) and the estimated number of driver substitutions in a list of 723 putative cancer genes across 560 breast cancer whole-genomes. A total of 2,786 coding substitutions are found in these genes across the 560 patients (data from Nik-Zainal et al., 2016). Of these, 579 were annotated as likely driver mutations by a careful and conservative manual curation in the original publication (Nik-Zainal et al., 2016) (blue bar). Using the trinucleotide dN/dS model on this dataset, restricted to these 723 genes, yielded a global dN/dS for all non-synonymous substitutions of 1.42 (CI95%: 1.29, 1.58). Reassuringly, this led to an estimated number of drivers consistent with the manual annotation: 668.9 (CI95%: 507.5, 815.3). Error bars depict 95% CIs. (B) Scatterplot of the estimated average number of coding driver substitutions per tumor in 369 known cancer genes and in all genes of the genome. This is a scatterplot representation of the bottom panels of Figures 4A and 4B, to emphasize the extent of coding driver substitutions occurring outside of the list of 369 cancer genes. Error bars depict 95% CIs. Note that the two cancer types whose estimates appear under the diagonal (mesothelioma –MESO- and thymoma –THYM-) have CIs extending above the diagonal, as expected. (C) Number of driver coding substitutions per tumor by clinical stage (see STAR Methods for details and interpretation). The panels compare stage I and stage IV tumors for the datasets with available clinical annotation, using either dN/dS-based estimates of the numbers of drivers per tumor (top panel) or raw counts of non-synonymous mutations in known cancer genes (bottom panel). Briefly, no consistent and statistically significant differences were observed.

From an observed dN/dS ratio, we can estimate the number of extra non-synonymous mutations over what would have been expected under neutrality (Greenman et al., 2006). For example, combining all coding mutations observed in 369 cancer genes across 689 breast cancer samples yields dN/dS = 1.95 (CI_95%_: 1.72–2.21). This implies that there are 1.95× more non-synonymous mutations than expected neutrally, or, equivalently, 49% (CI_95%_: 42%–55%) of the observed non-synonymous mutations are positively selected driver mutations (Figure 4A). Although this calculation does not inform which of these mutations are drivers, it provides a statistical framework for inferring the fraction and the absolute number of drivers in a catalog of mutations. Interestingly, manual annotation of breast cancer genomes has led to very similar estimates of the number of driver mutations in known cancer genes per tumor (Figure S4A) (Nik-Zainal et al., 2016).

Estimation of the number of driver mutations per tumor using this approach requires an accurate calculation of dN/dS ratios, so we took additional cautionary steps. Small inaccuracies in the mutation model can lead to systematic biases in the estimated numbers of drivers, especially in patients with high mutation burden. We found that this was particularly problematic in melanoma where the mutation signature is known to have sequence context biases beyond the immediate 5′ and 3′ neighbors of the mutated base (Pleasance et al., 2010a). A mutation model based on the pentanucleotide sequence context considerably outperformed the trinucleotide model in melanoma. Reassuringly, for all other tumor types, estimates of the number of driver substitutions per tumor obtained under the trinucleotide and pentanucleotide models were strongly concordant (Figure S1E), indicating that any remaining, uncorrected substitution biases are unlikely to impact our results.

Estimated dN/dS values on 369 known cancer genes (Table S3) varied extensively across cancer types (Figure 4A). Using these ratios, we estimate that ∼75% of the non-synonymous mutations occurring in known cancer genes in low-grade glioma are driver mutations; this fraction is only ∼25% in melanoma, with other tumor types spanning this range. Combining these estimates with total mutation burden, we infer that the average number of coding substitutions in known cancer genes that are driver mutations ranges from <1/patient in sarcomas, thyroid, and testicular cancers and mesotheliomas to 3–4/patient in bladder, endometrial, and colorectal cancers (Figure 4A).

We can extend this analysis to all genes in the genome to provide the first comprehensive estimates of the total number of driver coding substitutions per tumor. Unlike simply counting the number of non-synonymous mutations seen in known cancer genes, this estimate is not constrained to known cancer genes and comprehensively measures the number of all coding driver substitutions per tumor. We find that the fraction of all coding mutations estimated to be drivers is low in most cancer types (Figure 4B). For example, only 5.0% (CI_95%_: 3.0%–6.9%) of non-synonymous coding point mutations in head and neck cancers are predicted to be drivers. Interestingly, the average number of coding substitutions per tumor that are driver mutations is consistently modest, typically around 4/tumor and ranging from 1–10/tumor across tumor types (Figure 4B). We note that this is an estimate of the average number of coding driver substitutions per tumor for each tumor type; the actual number for individual patients might vary extensively around this average. Estimates of the number of driver mutations per tumor based on all genes are approximately twice those from the 369 cancer genes, suggesting that about half of driver mutations occur in cancer genes yet to be discovered (Figures 4B and S4B).

The preceding estimates are limited to coding non-synonymous base substitutions. To estimate the numbers of small indels and synonymous substitutions that could be drivers, we measured the overall excess of these changes in known cancer genes by using putative passenger genes to estimate background mutation rates (Supek et al., 2014). Although these values are likely to be slight underestimates due to the small number of driver mutations hidden in undiscovered cancer genes, this will have minimal quantitative impact. Reassuringly, this more extensive model yielded very similar estimates for non-synonymous coding substitutions to these obtained above from dN/dS (Figure 4C). We find that indels appear to contribute a similar number of driver mutations as truncating substitutions (nonsense and essential splice site mutations), with an average of ∼0.7 coding indel drivers per tumor in the 369 known cancer genes. Furthermore, synonymous driver mutations are rare but not negligible (∼0.09 per tumor in known cancer genes), in agreement with previous studies (Supek et al., 2014). See STAR Methods for a more detailed evaluation of the distribution of synonymous mutations across known cancer genes.

To evaluate whether the number of driver mutations significantly increases in more advanced cancers, we generated independent estimates for stage I and stage IV tumors (Lee et al., 2015). Interestingly, no significant differences in the number of estimated drivers or in the overall number of nonsynonymous mutations per tumor in known cancer genes were observed between early and metastatic tumors (Figure S4C).

### Gene-by-Gene, Histology-by-Histology Driver Mutations

Ultimately, if we are to use genomics to underpin precision medicine, an important step will be to infer which mutations in a given patient are drivers. As we have seen, not all somatic mutations in a given cancer gene are drivers, but dN/dS offers a framework to estimate these probabilities. We find that across tumor suppressor genes, whether missense substitutions are likely to be drivers or not varies considerably. For example, the tumor suppressors *ARID1A*, *RB1*, and *APC* show dN/dS values for missense mutations close to one suggesting that the vast majority of missense mutations seen in these genes across all cancers are genuinely passengers, even though >95% of observed truncating mutations are estimated to be drivers (Figure 4D). In contrast, the dN/dS value for missense mutations in *TP53* indicates that >95% of the missense mutations observed in this gene are drivers.

Such analyses highlight important differences across tumor types in the distribution of driver mutations. For example, in breast cancer, virtually all nonsense substitutions and ∼90% of missense substitutions in *PTEN* are driver mutations. However, in clear cell kidney cancer, only nonsense mutations in *PTEN* are significantly enriched, with no significant excess of missense substitutions above expectation. In lung adenocarcinoma, neither missense nor nonsense substitutions in *PTEN* were significantly more recurrent than expected, despite this cohort having good statistical power. Similarly, for oncogenes, we estimate that >10% of missense substitutions in *PIK3CA* in lung adenocarcinomas are passenger mutations, whereas only 1%–2% of such events in breast cancer are (Figure 4D).

### Hypermutator Tumors

The analyses above (Figures 4A–4C) were restricted to tumors with fewer than 500 coding mutations/tumor (<17 mutations/Mb), comprising 92% of samples in the cohort. A minority of tumors display a hypermutator phenotype, and their selection landscape remains poorly understood. In particular, it is unclear whether hypermutation leads to a higher number of driver mutations in a tumor or simply allows a clone to acquire a fixed complement of drivers faster than competing clones. All methods for detecting selection rely on models of the background mutation process, and even very small inaccuracies in these models can lead to considerable biases in samples with large numbers of mutations.

We first evaluated how the number of driver mutations changes with increasing mutation rates in tumors with burdens up to 1,000 coding substitutions. We find that as mutation burden increases, the dN/dS ratio converges toward 1 (Figure 5A). When this is used to estimate the number of coding substitutions that are drivers, we find that there is a sublinear relationship—as mutation burden increases, so too does the number of drivers, but with ever decreasing rates. This implies that the overall number of driver mutations per tumor does increase with total mutation burden, even though they represent an ever-smaller proportion.

Hypermutator tumors can be classified according to their dominant mutation process, which manifests as specific mutational signatures (Alexandrov et al., 2013). Using signature decomposition, we classified tumors with >1,000 coding mutations into five classes (Figure 5B). In tumors characterized by APOBEC mutagenesis, tobacco exposure, or mismatch repair deficiency, dN/dS ratios were very close to 1, confirming that the vast majority of coding mutations in these tumors are passengers (Figure 5C). However, there were significant biases in dN/dS away from 1 in *POLE* hypermutators (signature 10) and in tumors dominated by UV-induced mutations (signature 7). For UV-induced mutations, using a pentanucleotide dN/dS model eliminates the bias, consistent with this mutational signature extending beyond the base immediately up- and downstream of the variant.

*POLE* hypermutator tumors harbor germline or somatic mutations in the exonuclease domain of DNA polymerase-ε, affecting its proofreading ability leading to vastly increased mutation rates (Church et al., 2013, Rayner et al., 2016). Mutations mostly occur at three specific trinucleotides, generating a dramatically increased proportion of nonsense mutations as well as enriching for particular amino acid substitutions (Rayner et al., 2016). We found that the local sequence context for mutations in *POLE* tumors was biased considerably beyond the trinucleotide context, extending at least 4 bases either side of the mutated base (Figure 5D). This extended sequence context would not be fully captured by the trinucleotide or even the pentanucleotide dN/dS model. To explore this further, we simulated a set of random, neutral, *POLE* mutations using the observed 9-base sequence context. The dN/dS ratios from this simulation closely approximate those observed in *POLE* tumors (Figure 5C), suggesting that the high frequency of missense and nonsense mutations reported in *POLE* hypermutators (Castro-Giner et al., 2015) is broadly what would be expected under neutrality.

The combination of these biases and the high numbers of mutations prevent accurate estimation of the number of driver mutations in hypermutators across the whole exome. However, approximate estimates for the number of driver substitutions in known cancer genes can be derived by correcting dN/dS estimates in cancer genes by those seen in likely passenger genes. Reassuringly, this correction has little impact on our estimates in non-hypermutators (Figure 5E), suggesting that this approach is not overly conservative. In hypermutators, the analysis suggests that only a small fraction of missense substitutions in known cancer genes are drivers (Figure 5F) and that the absolute number of driver mutations per tumor in these genes is modest (Figure 5G). Interestingly, even after correction, *POLE* tumors show a considerable excess of nonsense mutations in known cancer genes when compared to likely passenger genes, with ∼20% of them predicted to be drivers, equating to ∼4–8 per tumor. Consistent with this prediction, a fifth of the nonsense mutations observed in the 369 cancer genes occurred in just 8 tumor suppressor genes (*APC*, *ATM*, *PTEN*, *MGA*, *PIK3R1*, *ARID1A*, *NF1*, and *FAT1*)—all target genes expected for the tumor types where *POLE* mutations arise, namely colorectal and endometrial cancers.

Taken together, these data suggest that hypermutator tumors usually acquire more driver mutations than those with lower mutation burdens, although the increase is proportionally much smaller than the increase in mutation rate.

## Discussion

By adapting methods from evolutionary genomics and applying them to thousands of cancer genomes and to five healthy tissues, we have observed a universal pattern of selection in somatic evolution, characterized by a dominance of positive over negative selection. We have found that negative selection is a surprisingly weak force during cancer development, which in turn has allowed us to obtain the first exome-wide genetic estimates of the number of driver coding substitutions across a range of tumor types.

The absence of negative selection on coding point mutations in cancer is remarkable, especially because it is the predominant evolutionary pressure in the germline. Clearly, the vast majority of genes are dispensable for any given somatic lineage, presumably reflecting the buffering effect of diploidy and the inherent resilience and redundancy built into most cellular pathways. This helps explain why cancers can tolerate extreme levels of hypermutation, evidenced by tumors that acquire many hundreds of mutations with every cell division (Shlien et al., 2015). Our results also suggest that negative selection on point mutations is largely absent during normal somatic tissue maintenance as well. This has important implications for the somatic mutation theory of aging (Morley, 1995), because it would argue that point mutations deleterious to the carrying cell do not drive cellular senescence, exhaustion, and death. Rather, if point mutations do play a role in aging of somatic tissues, it will be through the functional consequences to the organism of mutations that are selectively neutral or advantageous to the clone.

The conceptual framework we have developed for directly enumerating the excess or deficit of mutations with respect to the neutral expectation could be adapted to explore the role of driver mutations in non-coding regions of the genome. Furthermore, with increasing numbers of tumors being sequenced, we will be able to deploy such reasoning at ever higher resolution to estimate probabilities that variants in particular exons or domains of a gene in a particular tumor type are driver mutations. Such approaches could ultimately underpin statistically rigorous, personalized annotation of driver mutations, a crucial step in successfully implementing precision oncology.

## STAR★Methods

### Key Resources Table

**Table:** 

REAGENT or RESOURCE	SOURCE	IDENTIFIER
**Software and Algorithms**		

Algorithm and software for measuring dN/dS in cancer genomes	Cancer Genome Project, Wellcome Trust Sanger Institute	https://github.com/im3sanger/dndscv
Algorithms for calling somatic mutations – ASCAT (copy number)	Cancer Genome Project, Wellcome Trust Sanger Institute	Raine et al., 2016
Algorithms for calling somatic mutations – CaVEMan (substitutions)	Cancer Genome Project, Wellcome Trust Sanger Institute	http://cancerit.github.io/CaVEMan/
Algorithms for calling somatic mutations – Pindel (indels)	Cancer Genome Project, Wellcome Trust Sanger Institute	http://cancerit.github.io/cgpPindel/

**Other**		

BAM files for TCGA exomes	TCGA	https://cancergenome.nih.gov/

### Contact for Reagent and Resource Sharing

Further information and requests for resources and reagents should be directed to and will be fulfilled by the Lead Contact, Iñigo Martincorena (im3@sanger.ac.uk).

The BAM files used in these analyses were generated by and downloaded from TCGA. Due to restrictions on their use, we are not allowed to redistribute the files, but they can be accessed from source by researchers who have obtained the appropriate approvals.

### Experimental Model and Subject Details

Paired tumor and normal exome sequencing files from 9,699 cancer patients were downloaded from CGHub between November and December 2015. The samples, sequenced by TCGA (https://cancergenome.nih.gov/), correspond to 29 different tumor types. Colon and rectal cancer were grouped together as colorectal cancer. Table S1 shows the list of cancer types used in this study, their TCGA 4-letter code names, the longer abbreviations used in this study and the number of samples eventually selected for analysis (7,664 across all cancer types).

### Method Details

#### Calling of point mutations and indels

The data were uniformly reprocessed using the Wellcome Trust Sanger Institute’s variant calling algorithms to ensure uniformity across cancer types and to have control over the filtering of mutations at polymorphic sites. Owing to negative selection during human evolution, germline polymorphisms are heavily enriched in synonymous substitutions (Figure 1A). As a consequence, incomplete removal of germline polymorphisms from the collections of somatic mutations can lead to an underestimation of dN/dS ratios, while removal of genuine somatic mutations at polymorphic sites can lead to an overestimation of dN/dS ratios (see STAR Methods and Figures S1B and S1C for analyses on the impact of germline SNPs in catalogs of somatic mutations).

Paired-end reads were aligned to the reference human genome (GRCh37, hs37d5 build) using *BWA-MEM*. Substitutions were called using *CaVEMan* (Cancer Variants Through Expectation Maximization: http://cancerit.github.io/CaVEMan/) (Jones et al., 2016). Indels were called using cgpPindel v2.0 (http://cancerit.github.io/cgpPindel/) (Raine et al., 2015). A panel of unmatched normal samples (sequenced at the Wellcome Trust Sanger Institute) was used to remove common sequencing and mapping artifacts.

#### Quality controls and use of TCGA calls in five cancer types

Only pairs of samples with the same TCGA barcode ID to those used by TCGA in their public somatic mutation calls were considered for further study. To minimize the risk of germline polymorphisms in the collections of somatic mutations, somatic calls at sites with less than 10 reads of sequencing coverage in the matched normal sample were excluded. To ensure that somatic calls from our pipeline were not excessively different from those released by TCGA, samples in which our algorithms called < 50% of the coding mutations publicly released by TCGA were excluded. Samples with > 3,000 coding mutations (*i.e.* ∼100 mutations/Mb), including substitutions and indels, were excluded from all of the analyses in this study. After applying these filters, a total of 7,664 samples were used for the analyses in this paper.

Comparison of the mutation calls obtained from our pipeline to those released by TCGA for the same samples suggested low sensitivity of our pipeline in five of the 29 cancer types analyzed: acute myeloid leukemia (LAML), kidney chromophobe (KICH), pheochromocytoma and paraganglioma (PCPG), prostate adenocarcinoma (PRAD) and pancreatic adenocarcinoma (PAAD). For these five cancer types, public TCGA mutation calls were used in this study instead of those from our pipeline. These five cancer types were used in the driver discovery analyses, since these analyses are largely robust to minor germline contamination or over-filtering at polymorphic sites. However, these cancer types were excluded from the analyses of negative selection (Figure 3) and the estimation of number of driver substitutions per tumor (Figure 4), where moderate biases to dN/dS can affect the interpretation of the results.

#### Calling of copy number changes

We used the ASCAT algorithm (Van Loo et al., 2010) to identify copy number changes across 13,241 TCGA samples using Affymetrix SNP6 arrays. CEL files provided by TCGA were processed using PennCNV libraries (Wang et al., 2007) to obtain logR and BAF values. The logR values were subsequently corrected for GC content to decrease wave artifacts, which often affect samples profiled by SNP arrays. Copy number profiles for all tumor samples were then inferred from the corrected data using ASCAT version 2.4.2 (Van Loo et al., 2010).

#### List of 369 known cancer genes

To quantify the fraction of non-synonymous substitutions observed in known driver genes that are genuine driver mutations, we used a list of 369 high-confidence driver genes (Figure 4A). This list was compiled by merging the list of 174 *COSMIC classic genes* from version 73 of the COSMIC database (Forbes et al., 2015), the list of 219 significantly mutated genes reported by Lawrence et al. (2014) and the list of 204 genes identified as significantly mutated by the present study. The full list of 369 known cancer genes is available in Table S3.

### Quantification and Statistical Analysis

#### A dN/dS model for cancer genomics

dN/dS (also called Ka/Ks) is the ratio between the rate of non-synonymous substitutions per non-synonymous site and the rate of synonymous substitutions per synonymous site. First developed in the 1980s (Miyata and Yasunaga, 1980, Nei and Gojobori, 1986), it has a long history in the detection of negative and positive selection from sequencing data (Yang and Bielawski, 2000).

dN/dS is particularly suitable for the analysis of coding mutations in cancer genomes, for several reasons. First, unlike evolutionary comparisons of distant species, in which a change between two sequences may be the result of multiple changes to the site over the course of evolution, the density of substitutions per site in cancer is extremely low (typically < 10^−5^ mutations per site) (Martincorena and Campbell, 2015). This greatly simplifies the estimation of rate parameters and facilitates the development of more complex mutation and selection models (Greenman et al., 2006). Second, while some concerns exist regarding the use of dN/dS within a highly-recombining population (Kryazhimskiy and Plotkin, 2008), these considerations do not apply to somatic mutations accumulated in a cancer sample. That is both because cancer cells evolve asexually and because collections of somatic mutations are identified by comparing a cancer sample to the ancestral genome, rather than comparing two individuals or cells from a population. Finally, dN/dS offers a measure of selection largely free of assumptions, in contrast to population genetic tests of selection, in which apparent violation of neutrality can result from demographic changes rather than selection.

##### Poisson framework

In this study we adopt and expand upon the Poisson framework developed by Greenman et al. (2006). Mutations are classified according to their substitution type (*i*) (depending on the substitution model) and functional impact (synonymous –*s*-, missense –*m*-, nonsense –*n*- and essential splice sites –*e*-). Note that, throughout the paper, the term “truncating substitutions” refers to nonsense and essential splice site substitutions together. For example, the number of C > T synonymous mutations (*n*_C > T,s_) in a collection of samples is modeled as a Poisson process:

$$n_{C>T,s}∼Poisson\left(\lambda =t\;r_{C>T}L_{C>T,s}\right)$$

Where *t* is the density of substitutions per site, *r*_C > T_ is the relative rate of C > T substitutions per site, and *L*_C > T,s_ is the number of C sites in which a C > T change is synonymous. In this parameterization, one rate parameter of the substitution matrix is arbitrarily set to 1 (*e.g*. r_G > T_ = 1, so that all other rates are relative rates with respect to it). For non-synonymous sites, an extra parameter reflects the effect of selection on the accumulation of mutations:

$$n_{i,m}∼Poisson\left(\lambda =t\;r_{i}L_{i,m}\omega_{m}\right)$$

$$n_{i,n}∼Poisson\left(\lambda =t\;r_{i}L_{i,n}\omega_{n}\right)$$

$$n_{i,e}∼Poisson\left(\lambda =t\;r_{i}L_{i,e}\omega_{e}\right)$$

The ω parameters are the dN/dS ratios inferred by the model after correcting for the rates of different substitution classes (*r*_i_) and for sequence composition (*L*). Maximum-likelihood estimates for all parameters in the model can be efficiently obtained by Poisson regression.

Although a Poisson implementation of dN/dS is particularly suitable for cancer genomic data, it can similarly be used in other resequencing studies, especially as long as the density of mutations per site is low. This includes, for example, studies of human evolution and bacterial populations.

##### Substitution models

The simplest dN/dS implementations, such as the Nei-Gojobori model (Nei and Gojobori, 1986), treat all substitutions as a single substitution class. More sophisticated likelihood-implementations, widely used nowadays, instead use a substitution model with two substitution classes: transitions (C < > T, A < > G) and transversions (C < > A, C < > G, T < > A, T < > G) (*i.e.* they use a transition/transversion ratio as a single rate parameter) (Goldman and Yang, 1994). More complex mutation models include the GTR (General Time Reversible) model with 6 mutation classes, one for each of the 6 possible reversible base changes.

Somatic mutations in cancer have been shown to display strong context-dependence, particularly from one base upstream and downstream of the mutant base (Alexandrov et al., 2013). As we show in STAR Methods and Figure S1A, the use of simplistic substitution models can lead to severe systematic under- or over-estimation of dN/dS ratios and erroneous inference of selection. Previous studies of selection in cancer genomics have accounted for only some of this context-dependence, especially the high rate of C > T at CpG dinucleotides (Greenman et al., 2006, Lawrence et al., 2013, Yang et al., 2003).

In this study, to comprehensively avoid biases emerging from context-dependent effects from one base upstream and downstream of the mutant base, we use a full trinucleotide model with 192 rate parameters, one for each of the possible trinucleotide substitution rates. By using a model with 192 rates, as opposed to 96 rates, we accommodate the possibility of strand asymmetry emerging from transcription coupled repair in coding regions (Pleasance et al., 2010b). More complex models, including a full pentanucleotide substitution model, were also evaluated for specific applications (see STAR Methods and Figures S1D and S1E).

The adequacy of different substitution models in molecular evolution is often evaluated using Likelihood-Ratio Tests, Akaike Information Criterion (AIC) or Bayesian Information Criterion (BIC). In all 29 cancer types studied here, using AIC the fit of the substitution model with 192 parameters was vastly superior to a model of 12 parameters without context-dependence. For example, AIC values for the breast cancer dataset used in this study were 3,689.6 and 39,750.3 under the models with 192 and 12 parameters, respectively.

##### Modeling variable mutation rates across genes: dNdScv

In early exome studies with small numbers of samples, methods to detect significant mutation recurrence at gene level often assumed that the substitution rate was uniform across genes (Bolli et al., 2014, Greenman et al., 2007, Lawrence et al., 2013). In the Poisson framework described above, this is achieved by having a single *t* parameter shared across all genes (model called ***dNdSunif***). Maximum-likelihood estimates for the parameters across genes (*t*, *r*_i_, ω_m_, ω_n_ and ω_e_) are obtained by Poisson regression.

However, mutation rates are known to vary substantially across genes and models assuming uniform mutation rates across genes lead to the identification of large numbers of false positives when applied to relatively large numbers of samples (Lawrence et al., 2013). A simple way to avoid this problem is to have a separate *t* parameter for each gene (model called ***dNdSloc***). This is similar to most dN/dS implementations used in comparative genomics, in which the background mutation rate in a gene is directly estimated from the number of synonymous mutations observed in the gene. Although we have used this model successfully in cancer genomic datasets containing thousands of samples (Wong et al., 2014), it lacks statistical power to detect positively selected genes in smaller datasets.

The mutation rate is known to vary across genes depending on their expression level, replication time and chromatin state (Lawrence et al., 2013, Pleasance et al., 2010a, Polak et al., 2015, Schuster-Böckler and Lehner, 2012). Some methods designed to identify recurrently mutated genes in cancer genomes, exploit this knowledge to improve their background mutation rate models. For example, *MutSigCV* uses three covariates to estimate the mutation rate of each gene, by using information from other genes with similar covariate values (Lawrence et al., 2014, Lawrence et al., 2013). Inspired by this work, we developed ***dNdScv***, a method that combines dN/dS with a negative binomial regression on a large number of covariates.

We model the variation of the normalized mutation rate per base pair (*t*) across genes as following a Gamma distribution. In a given dataset, the observed number of synonymous mutations per gene –*j*- (*n*_s,j_) can then be modeled as a Poisson process whose mean is drawn from a Gamma distribution reflecting the variation of the mutation rate across genes.

$$n_{s,j}∼Poisson\left(\lambda \right)$$

λ∼Gamma(α,β)

Since the negative binomial distribution is a Gamma-Poisson compound distribution, the number of synonymous mutations per gene is modeled as following a negative binomial distribution. This enables the use of a negative binomial regression framework to estimate the background mutation model across genes for each dataset. Gene size, gene sequence and the impact of the substitution model are all accounted for as an offset in the model (reflecting the *exposure* of the gene). The normalized mutation rate per site, *t*, is modeled as Gamma-distributed across genes, reflecting the uncertainty in the variation of the mutation rate across genes remaining after accounting for the exposure of the gene. Covariates can then be used in this framework, to improve the estimated background rate for a gene and reduce the unexplained variation of the mutation rate, and so reduce the dispersion of the underlying Gamma distribution. A reduction in the unexplained variation of the mutation rate leads to more sensitivity for the detection of selection, while the use of overdispersion in the form of the Gamma distribution, reflecting the uncertainty in mutation rates across genes, ensures good specificity.

In R code, the regression is performed using:

model=glm.nb(n_syn∼offset(log(expected_syn))+covariate_matrix)

where:

*n_syn* for gene *j* is: *n*_s,j_ = $\sum_{i}n_{i,s,j}$

*expected_syn* for gene *j* is: *E*_s,j_ = $t\sum_{i}r_{i}L_{i,s}$ (with ***t*** being constant across genes).

This framework allows to use a large number of covariates and variable selection approaches to improve the background mutation rate model. In this study, we have used as the covariate matrix the first 20 principal components of 169 chromatin marks from the RoadMap Epigenomics Project (Kundaje et al., 2015). This included data from 63 cell lines and 10 different epigenetic marks (H3K9me3, H3K36me3, H3K27me3, H3K4me1, H3K4me3, H3K9ac, H3K23ac, H3K14ac, H2AK9ac and DNase). Since it has been shown that epigenomic landscapes derived from cell lines more closely related to a cancer type are better predictors of its local mutation density (Polak et al., 2015), there is added value in using a wide set of epigenomic covariates. The use of a regression framework hence allows to build complex and fully data-driven background mutation models for each dataset.

The negative binomial regression estimates a Gamma distribution for the uncertainty on *t*_j_ after considering the gene size, the gene sequence, the substitution model and the covariates. Hence, the likelihood for *t*_j_ can now be constrained both by the global knowledge of how the mutation rate varies across genes and the local number of synonymous mutations in the gene.

$$L\left(t_{\text{j}}\right)=L_{Poisson}\left(t_{\text{j}}|n_{\text{s},\text{j}}\right)L_{Gamma}\left(t_{\text{j}}|\text{α},\;\text{β}\right)$$

By using this joint likelihood, *dNdScv* weighs the amount of information on the mutation rate of the gene (Figures S2D–S2G). In small datasets, in which most genes have zero or a few synonymous mutations, the Gamma function dominates the likelihood (Figures S2E and S2G). In large datasets with sufficient numbers of synonymous mutations per gene, the Poisson function dominates and the *dNdScv* model converges to the *dNdSloc* model (see Figures S2E and S2G for examples).

Derivation of the expression for $L\left(t_{\text{j}}\right)$, $\left(dL/dt_{j}\right)=0$, gives a simple analytical solution for the maximum likelihood estimate of *t*_j_ under both the Poisson and Gamma constraints. The maximum likelihood estimate for the expected number of synonymous mutations in a gene under the *dNdScv* model (*E’*_s,j_) is: $\hat{E_{s,j}^{\prime }}=\hat{t_{j}}\sum_{i}r_{i}L_{i,s}=\left(n_{s,j}+\alpha -1/1+\beta_{j}\right)$. Where α and β_j_ are the shape and rate (inverse of scale) parameters of the Gamma distribution respectively, defined as: α = θ and β_j_ = *θ/μ*_j_ (μ_j_ is the predicted number of synonymous mutations for gene *j* according to the negative binomial regression model and θ is the overdispersion parameter of the regression model).

CIs for ω parameters under the *dNdScv* model (as used in Figure 4D) were obtained by profile likelihood integrating out *t*_j_.

##### Likelihood ratio tests for the inference of selection

In all three dN/dS models (*dNdSunif*, *dNdSloc* and *dNdScv*), inference of selection is performed using Likelihood Ratio Tests, similarly to traditional likelihood dN/dS models used in phylogenetics (Goldman and Yang, 1994, Yang and Bielawski, 2000). Examples of null and alternative hypotheses for different tests are shown below.

Global test for selection with free ω parameters (3 degrees of freedom):

$$\begin{array}{c} {\text{H}}_{0}:\omega_{\text{m}}=1;\omega_{\text{n}}=1;\omega_{\text{e}}=1\\ {\text{H}}_{1}:\omega_{\text{m}}\neq 1\text{;}\omega_{\text{n}}\neq 1;\omega_{\text{e}}\neq 1 \end{array}$$

Global test for selection with a single ω parameter for truncating substitutions (nonsense and essential splice site mutations) (2 degrees of freedom). This is the test used in the screen for positively selected genes in this study as it tends to be more sensitive than the fully unconstrained model above.

$$\begin{array}{l} {\text{H}}_{0}:\omega_{\text{m}}=1\text{;}\omega_{\text{n}}=1;\omega_{\text{e}}=1\\ {\text{H}}_{1}:\omega_{\text{m}}\neq 1;\omega_{\text{n}}=\omega_{\text{e}}\neq 1 \end{array}$$

Test for selection on missense mutations (1 degree of freedom).

$$\begin{array}{c} {\text{H}}_{0}:\omega_{\text{m}}=1;\omega_{\text{n}}\neq 1;\omega_{\text{e}}\neq 1\\ {\text{H}}_{1}:\omega_{\text{m}}\neq 1;\omega_{\text{n}}\neq 1;\omega_{\text{e}}\neq 1 \end{array}$$

Multiple testing correction is performed using Benjamini and Hochberg’s false discovery rate for all genes tested. To boost the statistical power to detect selection on known cancer genes, we use *restricted hypothesis testing* on an a priori list of known cancer genes, as described before (Lawrence et al., 2014). In this study, we use the list of 174 *COSMIC classic genes* from version 73 of the COSMIC database (Forbes et al., 2015) for RHT in the positive selection screen, and a list of essential genes for RHT in the negative selection screen.

##### Recurrence of insertions and deletions

dN/dS can be used to detect and quantify selection on coding substitutions, but not on small insertions or deletions (indels). To identify genes recurrently affected by indels or by other mutation types, such as dinucleotide substitutions or complex substitutions, we use a different model.

Briefly, a simple negative binomial regression model is used to estimate the expected rate of indels per gene. The length of the CDS of each gene is used as an offset and the 20 epigenomic covariates used in *dNdScv* are also used as covariates here. To minimize the risk of driver indels inflating the background model, known cancer genes are excluded when fitting the negative binomial model (in this study we used the list of 558 cancer genes in the *Cancer Gene Census* version 73 (Forbes et al., 2015). Applying this regression model to all genes in the genome provides an estimate of the mean indel rate expected in each gene and of the overdispersion of the model (θ). A *P*-value for the observed number of indels in each gene (*n*_i,j_) can be obtained using the cumulative negative binomial distribution. For each gene, we used Fisher’s method to combine the *P*-value from the indel model with the *P*-value obtained from *dNdScv* (with 2 degrees of freedom) for selection on coding substitutions. The resulting global *P*-value was used to identify genes under positive selection in Figure 2 and Table S2.

In this study, we tested two different implementations of the indel model: (1) considering the total number of indels per gene, or (2) considering the number of unique indel sites per gene (*unique-sites model*). The latter was designed to protect against recurrent indel artifacts and indel hotspots, such as microsatellites. This was motivated by the observation that genuine cancer genes most often show indels scattered throughout their sequence while some passenger genes occasionally show a high rate of indels at a single site, either due to artifacts or mutational hotspots. The performance of the two models was compared by running *dNdScv* on the pancancer dataset with both models. The *unique-sites* model was clearly superior, leading to much lower overdispersion across genes and a very high enrichment of *Cancer Gene Census* genes (64% of significant genes using the *unique-sites* model were CGC genes versus merely 8% when using the total number of indels per gene). The high overlap with the CGC also emphasizes the good performance of the indel model despite its simplicity.

We also tested the performance of the indel model in the presence of tumors displaying microsatellite instability (MSI). MSI tumors characteristically have a very high rate of indels at microsatellites due to DNA polymerase slippage that is left unrepaired in mismatch repair (MMR)-deficient tumors. To directly quantify the impact of MSI tumors, we repeated the search of driver genes in the pancancer dataset excluding MSI samples. To do so, we classified samples as microsatellite-stable (MSS) or MSI using the annotation provided in (Hause et al., 2016). 4,536 samples had an MSI/MSS status annotation, of which 127 were annotated as MSI. Although MSI tumors comprised less than 3% of the pancancer samples, they contributed 16% of all substitutions and 41% of all indels in the cohort. Despite their large contribution to the total number of indels, excluding MSI tumors from the analysis yielded nearly identical lists of driver genes, with differences only on genes close to the limit of significance, as expected (Figure S2C). Remarkably, this is the case even for the list of significant genes obtained when using only indels (Figure S2C), which might be expected to be much more severely affected by the removal of MSI samples contributing 41% of all indels in the dataset. This shows that the *unique-sites* indel model is robust to MSI tumors despite its simplicity and suggests that the vast majority of indels in MSI samples are likely passengers, consistently with our results on other forms of hypermutation (Figure 5).

#### Screen for positive selection at gene level (driver gene discovery)

To identify genes under significant positive selection we ran *dNdScv* on every cancer type separately and on all 7,664 samples together. *P*-values were calculated and adjusted for multiple testing using Benjamini and Hochberg’s false discovery rate. On inspection of the results, a small number of significant genes were found to be false positives resulting from recurrent sequencing or mapping artifacts in the collections of somatic mutations. To systematically remove false positives due to recurrent artifacts, all mutations found in significant genes were subject to an *in-silico* validation (see below), false calls were removed and *dNdScv* rerun on the cleaned dataset.

Genes found as significant (q-value < 0.05) in each cancer type are depicted in Figure 2 and in Table S2. Since combining results from multiple tumor types can inflate the global false discovery rate in the final list of significant genes, we then performed a global multiple testing correction on the entire matrix of *P*-values (20090 genes by 30 datasets) (as in Lawrence et al., 2014). This resulted in a list of 180 putatively positively-selected (driver) genes. Using restricted hypothesis testing led to the additional identification of 24 driver genes (Lawrence et al., 2014).

##### In-silico identification and removal of sequencing artifacts

Evaluation of significant hits revealed a small number of false positives due to recurrent artifacts that escaped our filters and our unmatched normal panel. To systematically identify recurrent artifacts leading to false positives in the screens for positive and negative selection, we used *ShearwaterML* (Gerstung et al., 2014, Martincorena et al., 2015).

*ShearwaterML* is a variant calling algorithm that relies on building a base-specific error model by using a large collection of unmatched normal samples. Sequencing artifacts caused by *Illumina* sequencing errors, PCR errors, DNA damage in a library, misalignment of reads or other causes, are expected to appear at similar frequencies in sequencing libraries of tumor or healthy (normal) tissue. Thus, all mutations identified in genes detected as significant by *dNdScv* were re-evaluated by *ShearwaterML*, comparing the number of reads supporting the mutation in the mutant sample to the frequency of errors seen across a large panel of TCGA normal samples from the same cancer type using a beta-binomial likelihood model (Martincorena et al., 2015).

To build a reliable panel of normal samples for each TCGA dataset and avoid filtering out genuine driver mutations, we excluded from the panels any normal sample with suggestive evidence of a mutation (> = 3 supporting reads) in a list of 344 recurrently mutated sites in known cancer genes. This reduces the risk of including samples in the normal panel with significant tumor contamination or hematopoietic clonal expansions (Xie et al., 2014).

*P*-values resulting from *ShearwaterML* were adjusted for multiple testing using Benjamini and Hochberg’s false discovery rate, correcting for n = *N*^∗^*S* tests to avoid a discovery bias (where *N* is the number of sites tested and *S* is the number of samples in each cancer type). Mutations with q-value > 0.20 were removed and *dNdScv* was re-run on the cleaned dataset. 49 genes were found to be heavily affected by artifacts, with more than 50% of the mutations found in them being considered artifactual by *ShearwaterML*. These genes were conservatively excluded from any significant hits in the positive selection screen.

The 49 genes heavily-affected by artifacts are: *AGAP10, AL445989.1, ANAPC1, ANKRD36C, AQP7, BMI1, C16orf3, CD209, CDC27, CDC7, CRIPAK, DTD2, EP400, FAM104B, FRG1, FRG1B, GNAQ, HLA-DRB5, HSPD1, IGBP1, KBTBD6, KRT14, KRT5, KRT6A, KRTAP1-5, KRTAP4-11, KRTAP4-3, KRTAP4-8, KRTAP4-9, KRTAP5-5, KRTAP9-9, MLLT3, MUC4, MUC8, NCOA6, PABPC1, PCDHB12, POTEC, POTEM, PPFIBP1, PRKRIR, PTH2, RGPD3, RGPD8, RP11-176H8.1, SLC35G6, TMEM219, TPT1* and *UBBP4*.

#### Negative selection analyses

##### Samples selected for negative selection analyses

As we have described, simplistic mutation models, germline contamination of the catalogs of somatic mutations and over-filtering of genuine somatic mutations at polymorphic sites can lead to biased dN/dS ratios. When analyzing dN/dS ratios close to 1, these biases can lead to wrong inferences about selection, as shown in Figures S1A–S1E and in STAR Methodss.

In order to avoid these biases, the analyses of negative selection shown in Figure 3 were carried out on a subset of all samples, encompassing 5,763 samples from 23 cancer types. First, the five cancer types with TCGA mutation calls were excluded from the analyses to have control over the filtering of germline mutations used during variant calling. Second, melanoma samples were excluded from these analyses since the mutation spectrum in melanoma causes a downward bias to dN/dS under the trinucleotide model (STAR Methods). Third, only samples with copy number information were included in the analyses, since this information was required for several of the analyses. Finally, only samples with fewer than 500 coding mutations per exome were included in the analyses to avoid hypermutator samples dominating the analyses and ensure representative results.

##### dN/dS distributions across genes

Observed dN/dS values at gene level are subject to considerable uncertainty due to the limited number of substitutions per gene. Hence, the variation in dN/dS values observed across genes (Figure 3A) is a composite of the true variation of selection across genes and Poisson noise in the counts of non-synonymous and synonymous mutations. Using mixture models, this technical variation can be eliminated to infer the underlying dN/dS distribution across genes.

For any gene, given a extent of selection (ω_m,j_) and a substitution model, the expected fraction of synonymous and missense substitutions in the gene can be calculated as follows: $\rho_{s,j}=\left(\sum_{i}r_{i}L_{i,s,j}/\sum_{i}r_{i}\left(L_{i,s,j}+L_{i,m,j}\omega_{m,j}\right)\right)$, $\rho_{m,j}=\left(\sum_{i}r_{i}L_{i,m,j}\omega_{m,j}/\sum_{i}r_{i}\left(L_{i,s,j}+L_{i,m,j}\omega_{m,j}\right)\right)$, respectively. The analysis of truncating substitutions (nonsense and essential splice site mutations) was done analogously.

##### Neutral simulations

To study how much variation in observed dN/dS values across genes is expected by simple noise under perfect neutral evolution, we first carried out a simple simulation. Using the expected fraction of synonymous and missense mutations per gene under neutrality (ρ_s,j,neutral_ and ρ_m,j,neutral_ given ω_m,j_ = 1 for all genes), and the total number of mutations observed per gene (*n*_s,j_+*n*_m,j_), we performed a random binomial simulation of the number of missense mutations per gene: $n_{m,j,random}∼B\left(n=n_{s,j}+n_{m,j},\;p=\rho_{m,j,neutral}\right)$. This yields a maximum-likelihood point estimate for dN/dS per gene of: $\left(n_{m,j,random}\rho_{s,j,neutral}\right)/\left(n_{s,j,random}\rho_{m,j,neutral}\right)$. This simulation revealed that most of the apparent variation observed in dN/dS across genes was technical, caused by the limited number of mutations per gene (Figure 3A).

##### Binomial mixture model

We can go beyond neutral simulations and infer the extent of the biological variation of ω across genes. Given ρ_s,j_ and ρ_m,j_ (as a function of ω_m,j_) and the total number of mutations seen in the gene (*n*_s,j_+*n*_m,j_), the probability of observing *n*_m,j_ mutations in a gene follows a binomial distribution. The advantage of using a binomial distribution contingent on the total number of observed mutations is that it makes the approach unaffected by the uncertainty in the background mutation rate of the gene.

$$P\left(n_{s,j},n_{m,j}|\omega_{m,j}\right)=\left(\begin{array}{c} n_{s,j}+n_{m,j}\\ n_{m,j} \end{array}\right)\rho_{m,j}^{n_{m,j}}\rho_{s,j}^{n_{s,j}}$$

We can extend this to model ω as a distribution across genes, for example using a discrete mixture model or integrating over a continuous distribution for ω. This is similar to existing approaches for modeling the distribution of ω across codons of a protein in comparative genomics (Nielsen and Yang, 1998). In this study, to avoid imposing a restrictive parameterization of the distribution of ω (dN/dS) across genes, we used a flexible discrete distribution with a fine grid. For the results shown in the main text, we used a discrete distribution defined as *ω ∈* (0, 0.1, 0.2, 0.3, 0.4, 0.5, 0.6, 0.7, 0.8, 0.9, 1, 1.1, 1.2, 1.3, 1.4, 1.5, 1.6, 1.7, 1.8, 1.9, 2, 3, 4, 5, 10, 15, 20), with a free probability mass function defined at these values ($\sum_{k=1}^{K}p_{k}=1$, where K is the number of points used in the discrete distribution and *p*_k_ the fraction of genes with *ω = ω*_*k*_). The global likelihood of the distribution of ω across genes can then be expressed as:

$$L\left(\omega \right)=\prod_{j=1}^{J}\sum_{k=1}^{K}p_{k}P\left(n_{s,j},n_{m,j}|\omega_{k,m,j}\right)$$

Where *J* is the total number of genes considered. Maximum likelihood estimates for the probability mass function $\left(\hat{p_{k}}\right)$ were obtained using an Expectation Maximization (EM) algorithm, initialized with uniform probabilities (*p*_k,0_ = 1/K). CIs were obtained by bootstrapping (sampling genes with replacement). Using distributions with more points yielded analogous results.

##### Estimation of the average number of mutations under positive and negative selection per tumor

Once the underlying ω distribution has been estimated $\left(\hat{p_{k}}\right)$, the probability that a gene has a particular value of ω can be calculated using the equation below. This equation corresponds to the posterior probability of a gene belonging to a ω class in the EM algorithm and it is identical to the empirical Bayes equation used for a similar purpose in the dN/dS literature (Nielsen and Yang, 1998).

$$P\left(\omega_{k,m,j}|n_{s,j},n_{m,j}\right)=\frac{\hat{p_{k}}P\left(n_{s,j},n_{m,j}|\omega_{k,m,j}\right)}{\sum_{i=1}^{K}\hat{p_{k}}P\left(n_{s,j},n_{m,j}|\omega_{i,m,j}\right)}$$

If a gene is evolving under a given value of ω_k,m,j_, the maximum likelihood estimate for the expected number of missense mutations in the gene, given the mutations observed, is: $\left(\left(n_{m,j}/\omega_{k,m,j}\right)+n_{s,j}\right)\rho_{m,j}$. If we assume that genes under positive selection (ω_k,m,j_ > 1) do not contain a significant number of sites under negative selection, or vice versa, we can use the value of ω_k,m,j_ to estimate the number of missense mutations fixed by positive selection or depleted by negative selection. Summing over all genes, we can obtain global estimates for the average number of missense mutations fixed by positive selection (δ_pos_) or depleted by negative selection (δ_neg_), per tumor.

$$\delta_{neg}=\frac{1}{N}\sum_{j=1}^{J}\sum_{\omega_{k}<1}P\left(\omega_{k,m,j}|n_{s,j},n_{m,j}\right)\left(1-\omega_{k,m,j}\right)\left(\frac{n_{m,j}}{\omega_{k,m,j}}+n_{s,j}\right)\rho_{m,j}$$

$$\delta_{pos}=\frac{1}{N}\sum_{j=1}^{J}\sum_{\omega_{k}>1}P\left(\omega_{k,m,j}|n_{s,j},n_{m,j}\right)\left(\omega_{k,m,j}-1\right)\left(\frac{n_{m,j}}{\omega_{k,m,j}}+n_{s,j}\right)\rho_{m,j}$$

Where *N* is the number of samples used in the analysis. CIs for these estimates were obtained by bootstrapping (sampling genes with replacement). It should be noted that, in the presence of both positive and negative selection acting on the same gene at different sites or in different samples, these estimates will underestimate the extent of positive and negative selection. However, in order to explain the observation that the vast majority of genes are estimated to have an average ω∼1 (Figure 3B), the combination of positive and negative selection should be nearly perfectly balanced across most genes in the genome, which is unlikely. This suggests that most genes seem to accumulate missense mutations largely neutrally and so that δ_pos_ and δ_neg_ are probably decent approximations.

In this study, we inferred $\hat{p_{k}}$, δ_pos_ and δ_neg_ for missense and truncating (nonsense and essential splice site) substitutions separately, as well as for three classes of mutations according to the copy number state of the region where the mutations occurred: haploid regions (1:0), loss of heterozygosity (LOH) regions with higher ploidy (n:0, with n > 1), and all others (*i.e.* regions without LOH). Estimates shown in Figure 3D include the sum of all of these mutation types. Figures 3A–3C show estimates for missense mutations in regions without LOH.

##### Gene-level analyses of negative selection

To identify whether any gene has a dN/dS value significantly lower than 1 we used a one-sided test on missense mutations alone:

$$\begin{array}{c} {\text{H}}_{0}:\omega_{\text{m}}=1;\omega_{\text{n}}\neq 1;\omega_{\text{e}}\neq 1\\ {\text{H}}_{1}:\omega_{\text{m}}\leq 1;\omega_{\text{n}}\neq 1;\omega_{\text{e}}\neq 1 \end{array}$$

This test has 1 degree of freedom and the resulting *P-*value from the Chi-square distribution is divided by two, as the test is one-sided. Since tissue-specific datasets lack statistical power to detect negative selection at gene level, we used the entire pancancer dataset (n = 7,664 samples) for this analysis. We used both the *dNdScv* model and *dNdSloc*. These tests did not find any gene under significant negative selection at false discovery rate < 10%.

To boost the statistical power on genes that may be suspected to be under stronger negative selection, we performed restricted hypothesis testing on an a priori chosen list of 1,734 essential genes. All genes yielded q-values higher than 0.10.

##### Power calculations

The power to detect negative selection in a gene (or a group of genes) under the *dNdSloc* model is determined by two main factors: (1) the effect size (the dN/dS ratio), and (2) the number of mutations in the gene (which is largely determined by the number of samples in the dataset, their mutation burden and the length of the gene). Under the *dNdScv* model, a third factor affecting the power is the uncertainty of the background model (*i.e.* the overdispersion of the negative binomial regression -θ-).

In order to study the power to detect negative selection under both models, we performed random simulations. Let ***m*** be the expected (average) number of coding mutations in a gene in a dataset, ρ_s_, ρ_m_ and ρ_t_ the fraction of synonymous, missense and truncating (nonsense and essential splice site) substitutions expected under neutrality, ω_m_ and ω_t_ the corresponding values of dN/dS, and α the shape parameter of the underlying Gamma distribution. Then we can simulate the number of synonymous, missense and truncating substitutions in the gene using:

$$m_{j}∼Gamma\left(\alpha =\theta ,\beta =\theta /m\right)$$

$$n_{s}∼Poisson\left(\lambda =m_{j}\rho_{s}\right)$$

$$n_{m}∼Poisson\left(\lambda =m_{j}\rho_{m}\omega_{m}\right)$$

$$n_{t}∼Poisson\left(\lambda =m_{j}\rho_{t}\omega_{t}\right)$$

*P*-values for two-sided tests under both *dNdSloc* and *dNdScv* can be calculated from these numbers (H_0_: ω_m_ = 1; H_1_: ω_m_≠1, df = 1). For each combination of ***m*** and ω tested, we performed 5,000 simulations. The fraction of *P-*values below 0.05 reflect the power to detect a gene as significantly under selection. The values used for ρ_s_, ρ_m_, ρ_t_ and θ are the average values for these parameters observed in the pancancer dataset (ρ_s_ = 0.287, ρ_m_ = 0.649, ρ_t_ = 0.064, θ = 6.03).

##### Group-level analyses of negative selection

Given the limited statistical power to detect negative selection at the level of individual genes, we searched for evidence of negative selection in groups of related genes. To do so, we first excluded a long list of 987 putative cancer genes, by combining gene lists from multiple sources. We then used the *dNdSloc* model to study selection on groups of genes, as defined by expression level, local chromatin state, essentiality and gene ontology functional annotation.

##### Expression

As a measure of the typical expression level of a gene across tumors, we calculated the mean of the log RSEM-normalized expression level of each gene across a collection of 6,190 TCGA samples (*.rsem.genes.normalized_results* TCGA files).

##### Chromatin state

As a measure of the typical chromatin state of a gene, we defined as heterochromatin and euchromatin those regions in which the six main ENCODE cell lines shared the same annotation.

##### Essentiality

As a list of genes essential for cell survival and growth, we used a collection of 1,734 core essential genes reported by a recent mutagenesis screen in haploid human cell lines (Blomen et al., 2015). This list of genes is heavily enriched in proteins participating in key cellular components and pathways, such as the ribosome, the spliceosome, the aminoacyl-tRNA biosynthesis pathway, the proteasome, RNA degradation, DNA replication, RNA polymerases and the cell cycle (Blomen et al., 2015).

##### Gene ontology

To search for evidence of negative selection at the level of functionally related genes, we used *Ensembl BioMart* to extract Gene Ontology (GO) annotations for all genes. To ensure adequate statistical power and reduce multiple testing correction, we only tested groups composed of at least 30 genes. We considered GO annotations of *Biological Processes*, *Cellular Components* and *Molecular Functions*. Overall we tested 1,242 functional groups of genes and performed Bonferroni multiple-testing correction (we used Bonferroni to account for the extensive overlaps between gene groups). Including all genes in the analysis yielded a large number of GO groups with evidence of positive selection on missense and/or nonsense substitutions (n = 428), but no group with evidence of negative selection. Excluding the long list of 987 putative driver genes dramatically reduced the number of functional gene groups with evidence of positive selection (n = 27), but still no GO group showed evidence of significant negative selection (Figure S3E). Repeating this analysis on mutations occurring in haploid regions did not identify any group of genes under clear negative selection.

#### Simplistic substitution models lead to biased dN/dS ratios and false inference of selection

Traditional implementations of dN/dS have typically used simplistic substitution models. The classic implementation of dN/dS by Nei and Gojobori (1986), for example, uses a substitution model in which all substitutions are equally likely (F81 substitution model). More sophisticated likelihood implementations of dN/dS, such as the widely used implementation in the *PAML* software package, typically use a simple substitution model with a different rate for all transitions (C < > T and G < > A changes) and all transversions (C < > A, C < > G, G < > C and G < > T changes) (HKY85 substitution model) (Goldman and Yang, 1994, Yang, 2007). A more complex substitution model, frequently used in molecular evolution but more rarely in dN/dS analyses, is the GTR (General Time Reversible) model, which has 6 mutation classes, one for each of the 6-possible reversible base changes (A < > C, A < > G, A < > T, C < > G, C < > T, G < > T).

In reality, the substitution rate often varies markedly depending on the exact nucleotide change and on the bases upstream and downstream of a base. This is particularly well understood in cancer, from the study of mutational signatures (Alexandrov et al., 2013). The use of simplistic mutation models is known to lead to biases in dN/dS estimates (Yang and Nielsen, 2000). While these biases may be of lesser importance in the presence of overwhelming negative or positive selection, they can have important implications when dN/dS ratios are close to 1, as is often the case in somatic evolution.

Figure S1A reveals how simplistic substitution models lead to systematic under or overestimation of dN/dS ratios and to wrong inference of selection. To generate this figure, the average trinucleotide substitution rates (192 parameters) were estimated in three different cohorts of samples, which are dominated by different mutational processes: pancancer (dominated by C > T mutations at CpG sites), melanoma (dominated by the UV-signature of C > T mutations at cytosines with a pyrimidine upstream) and lung adenocarcinoma (dominated by G > T mutations generated by tobacco smoking) (Alexandrov et al., 2013). Using the trinucleotide rates observed in each of these datasets, and the trinucleotide frequencies of the human exome, we simulated 100 datasets with 10,000 random coding substitutions per dataset. The correct dN/dS ratio in these simulations is 1, since the mutations were simulated entirely randomly, without selection. Figure S1A shows how estimated dN/dS ratios under different simplistic substitution models systematically deviate from the correct value of 1. In fact, these biases are large enough to suggest considerable negative and positive selection when using simplistic models.

These biases have important implications. For example, a study applying dN/dS to somatic mutations from breast cancer genomes used a Nei-Gojobori implementation of dN/dS (F81 substitution model), obtaining a global dN/dS∼0.82 (Ostrow et al., 2014). This led the authors to conclude that weak negative selection operates in cancer somatic mutations, when in reality this dN/dS ratio is a consequence of the downward bias in dN/dS under the Nei-Gojobori model (Figure S1A).

#### Impact of germline SNP contamination or SNP over-filtering

As shown in Figure 1A, coding germline SNPs are heavily enriched in synonymous mutations as a result of purifying selection on germline mutations during human evolution (dN/dS ratios for missense and truncating substitutions are 0.38 and 0.08, respectively). Identification of somatic mutations in cancer genomes requires careful removal of germline polymorphisms by sequencing a matched normal sample in addition to a tumor sample from each patient. Given the action of negative selection on germline SNPs, incomplete removal of SNPs from catalogs of somatic mutations can introduce a false signal of negative selection. To protect against germline SNP contamination, some pipelines systematically remove putative somatic mutation overlapping polymorphic sites in humans in addition to using a matched normal sample. However, since polymorphic sites are enriched in synonymous sites, such filtering strategy can lead to over-filtering of genuine somatic mutations, with a bias against synonymous sites.

Figures S1B and S1C show how both germline SNP contamination and over-filtering of SNP sites can have a considerable impact on global dN/dS ratios, resulting in signals of negative and positive selection, respectively. To generate this figure, we first simulated ten neutral datasets of somatic mutations by randomization of existing cancer genomic datasets. To these neutral datasets, we added 5% or 10% of randomly selected germline SNPs (Figure 1) or we subtracted any mutation overlapping known polymorphic sites using the dbSNP database.

Interestingly, this analysis confirms that global dN/dS ratios detect a very clear signal of negative selection even when only 5% of all mutations are germline SNPs. This further emphasizes the remarkable lack of negative selection reported in Figure 3, and in particular in Figure 3G after comprehensively removing known cancer driver genes.

SNP contamination and SNP over-filtering are likely to affect TCGA public somatic mutation calls from different datasets to different extents. This was apparent when we calculated global dN/dS ratios using the somatic mutation calls publicly released by TCGA. For example, the *COAD*, *READ* and *KICH* datasets showed significantly lower dN/dS ratios than expected: *COAD* = 0.92 (CI95%: 0.91, 0.94), *READ* = 0.91 (CI95%: 0.87, 0.95) and *KICH* = 0.94 (CI95%: 0.89, 1.00), suggesting the presence of SNP contamination in these datasets. To determine whether these low dN/dS ratios are truly caused by SNP contamination of the public catalogs of somatic mutations, we calculated the fraction of mutation calls overlapping common germline SNP sites (dbSNP database build 146). This revealed that these three datasets have a much higher fraction of somatic calls overlapping common dbSNP sites than other datasets, with 11.0%, 15.6% and 12.2% of all somatic mutation calls from TCGA overlapping known SNP sites (Figure S1C). In contrast, the median percentage of overlapping calls in all other cancer types from TCGA is 1.7% (range: 0.66%–3.3%).

Studies searching for evidence of negative selection based on public mutation calls from TCGA are likely to be affected by the confounding effects of SNP contamination and potentially SNP over-filtering. Having control over the strategy for SNP filtering was the main motivation for uniformly re-calling somatic mutations across TCGA datasets in the present study. In order to minimize the risk of germline SNP contamination, we required a minimum coverage of 10x in the matched normal sample of a putatively mutated site. To entirely avoid any risk of over-filtering of SNP sites that may introduce an upward systematic bias to dN/dS, we did not perform dbSNP filtering and all sites masked out by our unmatched normal panel were excluded from the calculation of available sites (*L*) in dN/dS. Reassuringly, *CaVEMan* somatic mutation calls for all TCGA datasets showed the expected low overlap with common dbSNP sites, with a median of 1.8% (range: 1.1%–3.2%), a figure consistent with the expectation from neutral simulations.

#### Cohort estimation of dN/dS without patient-specific substitution models

The dN/dS models described in this manuscript quantify mutation and selection at the level of a cohort of samples, not at the level of each individual tumor within a cohort. A single global substitution model is fitted to the mutations observed in a gene across multiple samples. Mutations in each sample could be modeled as individual Poisson processes, but since the sum of Poisson variables is also Poisson distributed, we can also model mutation and selection at a cohort level. As we show below, this has the advantage of enabling the use of more complex substitution models, avoiding the systematic biases associated with simplistic models. Fitting 192 trinucleotide rate parameters on a per-patient basis is clearly not possible from exome data, where most patients have fewer than 100 mutations, and so modeling mutations on a per-patient basis would normally require a compromise in terms of accuracy.

As we show below, the dN/dS estimates resulting from cohort-level estimation accurately reflect the mean selection pressure acting on the mutations in a gene and are unbiased by the presence of large heterogeneity in mutational signatures, burden and selection across the samples in a cohort. Before demonstrating this, it is important to clarify what dN/dS means at a cohort level. For example, in a gene subject only to negative selection, a dN/dS value of 0.8 in a cohort of samples means that 20% of all non-synonymous mutations in the gene observed across samples were lost by negative selection. This does not inform about whether negative selection mainly occurred in a subset of samples or in a subset of sites in the gene, but this information is not required for the claims of this study. When selection on a gene varies across samples, dN/dS is an unbiased estimate of the fraction of negatively selected mutations in a gene (or group of genes) across samples. Mathematically, dN/dS represents the weighted mean of selection across samples, weighted by the mutation rate of each sample (see below).

The validity of cohort-wide substitution models to estimate dN/dS accurately can be shown by simulation of mixtures of patients, even in the presence of wide heterogeneity in mutation rates, signatures and selection across patients. For example, we can simulate an extreme scenario of 100 samples, in which COSMIC’s signature 1 contributes all of the mutations in 50 patients and signature 5 all of the mutations in the other 50 patients (Alexandrov et al., 2013). Independently of whether all negative selection in a gene is spread out across samples or is concentrated on a subset of patients with a specific signature, cohort-based estimation of dN/dS without patient-specific modeling of mutational processes yields exact estimates of the fraction of all mutations that were negatively selected in each simulation. Figure S1F shows three extreme simulations:

1. *Simulation 1*: 100 patients, 50 dominated by signature 1 and 50 by signature 5, all with equal neutral mutation rate. dN/dS = 0.5 in all patients (i.e., randomly, 50% of all nonsynonymous mutations are removed from each patient).
2. *Simulation 2*: 100 patients, 50 dominated by signature 1 and 50 by signature 5, all with equal neutral mutation rate. dN/dS = 0 in 20 patients dominated by signature 1 and dN/dS = 1 in all other patients.
3. *Simulation 3*: 100 patients, 50 dominated by signature 7 and 50 by signature 3. All signature 7 patients have dN/dS = 0.1 and a burden of 100 coding mutations (before the action of selection). All signature 3 patients have dN/dS = 1 and a burden of 500 coding mutations.

In all cases, the global dN/dS estimated using a single substitution model for the entire cohort of samples accurately reflects the fraction of all nonsynonymous mutations in a gene removed by negative selection, which follows the equation: $f_{global}=\left(\sum_{j}b_{j}\omega_{j}/\sum_{j}b_{j}\right)$, with *b*_j_ being the mutation burden before selection in sample *j* and ω_j_ being dN/dS or the fraction of nonsynonymous mutations removed by negative selection in sample *j*.

#### Estimation of the number of driver mutations

##### Samples selected for the estimation of the number of driver mutations

All samples with *CaVEMan* mutation calls and less than 500 coding mutations per sample were included in this analysis, including the melanoma dataset. Overall, a total of 6,108 samples from 24 cancer types were included in the pancancer estimates of the number of driver mutations per tumor shown in Figure 4.

##### Estimating the number of substitutions fixed by positive selection from dN/dS

In the absence of negative selection and mutation biases, we can accurately estimate the number of mutations expected to have accumulated neutrally in a gene or group of genes. As described by Greenman et al. (2006), this can be used to estimate the number of mutations in excess that have been fixed by positive selection. Assuming a negligible role for negative selection, we can calculate the fraction (*f*_m_) and the absolute number (*d*_m_) of mutations in a gene or group of genes that are genuine driver mutations as:

$$f_{m}=\frac{\omega_{m}-1}{\omega_{m}};\;d_{m}=f_{m}n_{m};\;\forall \;\omega_{m}>1$$

In the presence of significant negative selection, these equations would provide lower bound estimates of the density and number of genuine driver mutations. However, our analyses suggest that negative selection has a small quantitative effect on the accumulation of passenger mutations in cancer. The same equations apply for nonsense and essential splice site substitutions.

Naively, one might expect that all non-synonymous mutations observed in a driver gene could have been positively selected and so that they could all be drivers. However, in the absence of negative selection, we should still expect passenger mutations to accumulate in driver genes at approximately the background rate predicted under neutrally and so the equations above are required to estimate the number of genuine driver substitutions. This is true even if a driver gene was under positive selection in every patient, as long as the extent of negative selection is negligible.

##### Pentanucleotide model and removal of polymorphic sites

We have shown that using an inadequate substitution model can lead to substantial biases in the estimation of dN/dS (Figure S1A; STAR Methods). Many applications of dN/dS do not require a very high accuracy, since true biological deviations from neutrality are often far larger than the biases caused by the substitution model. For example, identification of genes under positive selection in small datasets is often unaffected by the substitution model since dN/dS ratios of genuine driver genes can take very high values (see, for example, Figure 4D).

The estimation of the number of driver substitutions per tumor, however, requires accurate quantification of dN/dS ratios, since these ratios are often very close to 1. For example, the genome-wide dN/dS value for all non-synonymous substitutions in the pancancer dataset used in Figure 4B is 1.059 (CI_95%_: 1.052, 1.065). Given the proximity to 1, misestimating this value by a few percent would have a considerable impact on the estimates of the number of driver substitutions per tumor.

To minimize the risk of systematic biases in the estimation of genome-wide dN/dS values and the average number of driver substitutions per tumor shown in Figure 4, we took two additional precautions. First, we used a pentanucleotide context-dependent substitution model (3,072 rate parameters) instead of the trinucleotide model (192 rate parameters). Second, since somatic mutations called by our pipeline were filtered against an unmatched normal panel, common polymorphic sites in the human population (which are enriched in synonymous mutations) will be depleted of somatic mutations, which could lead to a very small upward bias in dN/dS. To entirely avoid this possible bias, all sites in the unmatched normal panel were excluded from the calculation of the numbers of synonymous and non-synonymous sites (*L*_i_) per gene.

##### Pentanucleotide model

The use of a full trinucleotide model comprehensively accounts for the majority of known context-dependent mutational biases. Previous studies suggest that context dependent effects beyond three nucleotides are relatively small (Alexandrov et al., 2013).

To evaluate the impact on dN/dS of context-dependent effects extending beyond one base up- and downstream, we compared whole-genome estimates of dN/dS across cancer types under the full trinucleotide (192 rate parameters) and a full pentanucleotide model (3,072 rate parameters). Figures S1D and S1E reveals that the addition of context-dependent effects beyond three nucleotides does not have a significant impact on genome-wide dN/dS ratios in any of the cancer types studied, with the exception of melanoma. This is due to UV-induced C > T mutations showing context-dependent effects extending beyond the trinucleotide level (Pleasance et al., 2010a).

With the exception of melanoma, in which the dominant mutation processes lead to a slight downward bias in dN/dS under the trinucleotide model, Figures S1D and S1E evidence that the trinucleotide model captures most of the relevant context-dependent effects required for a very accurate estimation of dN/dS in the cohorts studied here. *POLE* hypermutator tumors are another exception, as described in the main text (Figures 5C and 5D). Application of a pentanucleotide model to *POLE*-induced mutations, which show a wider context-dependence (Figure 5D), reduces the bias to dN/dS but does not solve it.

To avoid these biases, all analyses in Figures 4A and 4B, which require accurate dN/dS values, were carried out using the pentanucleotide substitution model.

##### Other mutation types: estimating the density of driver indels and synonymous mutations

Our estimates of the number of driver mutations per tumor using dN/dS are restricted to non-synonymous coding substitutions, including missense, nonsense and essential splice site substitutions. To obtain approximate estimates of the relative contribution of indels and synonymous substitutions to the number of driver mutations, we used a different approach not based on dN/dS. Briefly, the expected neutral rate of indels and synonymous substitutions on a collection of driver genes was estimated from their frequency on putative passenger genes, and this number was used to estimate the excess of these mutations observed in driver genes. This approach is conceptually analogous to the one used in (Supek et al., 2014) to estimate the frequency of synonymous driver mutations in cancer.

To estimate a background model for the *neutral* frequency of synonymous substitutions and indels we first excluded the long list of 987 putative cancer genes. We then used two separate negative binomial regression models for synonymous substitutions and indels. For synonymous mutations, we used as an offset the expected rate of synonymous substitutions per gene under the full trinucleotide model. Unlike the approach used in (Supek et al., 2014), this entirely avoids the confounding effect of variable sequence composition across genes and trinucleotide context-dependent mutational biases. For indels, we used the gene length as an offset. For both synonymous substitutions and indels, we used the 20 covariates described above to account for the regional variation of mutation rates across the genome. These models were then applied to the list of 369 high-confidence cancer genes to estimate the number of passenger indels and synonymous mutations expected to accumulate neutrally in these genes. This enables the calculation of observed/expected ratios for synonymous substitutions and indels in known cancer genes, and, analogously to using dN/dS ratios, the estimation of the fraction of these mutations that are genuine drivers and their absolute contribution to the number of driver mutations per tumor. CIs for these estimates were obtained by bootstrapping the number of mutations observed per gene.

To evaluate the reliability of this approach, we also applied it to missense, nonsense and essential splice site substitutions and compared the estimated number of driver mutations per tumor in known cancer genes to those obtained using dN/dS. As shown in Figure 4C, the estimates obtained from these two very different approaches are very consistent.

##### Identification of cancer genes with a higher frequency of synonymous mutations than expected

Although the vast majority of synonymous mutations observed in cancer genomes are passenger mutations and accumulate largely neutrally, our analysis and a previous study (Supek et al., 2014) suggest that a small number of them can act as driver mutations. We can use the negative binomial background model for synonymous substitutions described above to identify genes with an unexpectedly high density of synonymous mutations, in a similar way in which we identify genes recurrently mutated by indel drivers.

Running this analysis on the list of 369 cancer genes reveals that only *TP53* (q-value = 6.0e-6) and *CDKN2A* (q-value = 0.00058) have a convincing and statistically-significant higher than expected number of synonymous substitutions (q-value < 0.01). Close inspection of the mutations in *CDKN2A* revealed that the recurrent synonymous mutations observed are indeed truncating mutations affecting a different transcript of the gene, with a different reading frame, and so *CDKN2A* is not genuinely recurrently affected by synonymous driver mutations.

*TP53* has been previously reported to be the target of synonymous driver mutations (Supek et al., 2014), which affect the correct splicing of the transcript, and our analysis entirely supports this conclusion. The observed/expected ratio of synonymous substitutions in *TP53* is very high (∼6.8), which suggests that a majority of the synonymous mutations observed in *TP53* in our cohort of 24 cancer types are likely genuine driver mutations. In fact, in our cohort, over half of the synonymous substitutions observed in *TP53* affect the same site T125T (21 out of 39 synonymous substitutions in *TP53*), a recurrent synonymous hotspot known to lead to aberrant splicing (Supek et al., 2014). Hence, this single synonymous hotspot accounts for the majority of synonymous driver substitutions in *TP53*, although other synonymous mutations in *TP53* are also likely drivers.

A previous study identified a number of oncogenes with a higher density of synonymous mutations than expected by chance (Supek et al., 2014) and argued that these could be driver mutations affecting splicing. Among these genes, the study highlighted 11 oncogenes with a particularly high density of synonymous substitutions: *PDGFRA*, *EGFR*, *KDR*, *NTRK1*, *IL7R*, *TSHR*, *ELN*, *JAK3*, *ITK*, *GATA1* and *RUNX1T1*. This contrasts with our analysis, which only identified *TP53* as having a statistically-significant higher rate of synonymous mutations despite using a dataset with nearly twice as many samples as the dataset used in the previous study. An important difference between our analysis and that in the previous report is that our negative binomial model uses overdispersion to quantify the uncertainty in the estimated mutation rate for a gene. This makes our model more conservative, but also more robust against false positives caused by the neutral variation of the mutation rate across genes. We also control for trinucleotide sequence composition and trinucleotide mutation rates as well as 20 epigenomic covariates in the estimation of the background mutation rate per gene. Interestingly, even though both studies are based on TCGA samples and, in fact, share a large number of samples, only 4 of the 11 oncogenes highlighted in the previous study as having a high rate of synonymous mutations have observed/expected ratios of synonymous substitutions > 1.5x according to our model and none are considered significant under the negative binomial model (q-value > 0.5).

Overall, consistently with previous reports, our analyses suggest that certain synonymous mutations can indeed act as cancer driver mutations, of which the T125T hotspot mutation in *TP53* is probably the most striking example. However, there is little evidence that this is a general and frequent mechanism. Our analyses suggest that synonymous mutations contribute a small fraction (< 5%) of all driver mutations seen in cancer genomes (Figure 4C).

##### Correlation of number of drivers with tumor stage

Using the information on tumor stage for the TCGA cohort, we examined whether the estimated number of driver mutations was higher in more advanced or larger tumors. To do this, we fitted a linear mixed effects model with estimated number of drivers for each tumor type – stage combination as the dependent variable. The fixed effect was tumor stage, on a 1-4 scale, and we fitted a random effect for the intercept across tumor types. To allow for the variable precision in the estimate of the number of drivers for each tumor type – stage combination, we used inverse variance weighting from the CIs for each estimate of the number of drivers. Maximum likelihood estimation was used, and the hypothesis of whether stage correlated with number of drivers was evaluated using a likelihood ratio test. The R package *nlme* was used, and the line of code was:

drivers.by.stage.rand.intercept<−lme(num.drivers∼Stage,random=∼1|Tumourtype,weights=varFixed(∼Variance.num.driv.estimates),data=num.drivers.df,method=“ML”)

#### Replication strand bias

The full dN/dS trinucleotide model uses 192 parameters to describe all possible trinucleotide changes on either the transcribed or the untranscribed DNA strand. This accounts for transcriptional-strand asymmetry, often seen in somatic evolution because of processes like transcription-coupled repair (Pleasance et al., 2010a, Pleasance et al., 2010b).

A different form of strand asymmetry is replication-strand asymmetry, in which mutation rates differ in both strands depending on whether a strand is preferentially replicated by the leading or lagging strand of the replication fork. Such processes are very prevalent in certain mutational processes, such as microsatellite instability, APOBEC-induced mutations or *POLE*-induced mutations (Haradhvala et al., 2016). Accounting for this in our implementation of dN/dS is straightforward by using different rate parameters for the leading and lagging strands. Unfortunately, however, information on the preferential direction of replication is only available for a minority of genes in the genome (∼31% of genes) (Haradhvala et al., 2016). This complicates the use of replication strand in dN/dS in a systematic way.

Nevertheless, we can explore the impact of accounting for replication strand bias using those genes with available annotation. To do so, we classified genes into two classes according to whether the coding strand was preferentially replicated by the leading or lagging strands. Calculating dN/dS (using the 192-parameter trinucleotide model) separately on each class of genes accounts for replication and transcription strand biases simultaneously. These results can be combined for FDR adjustment and compared to the results obtained analyzing both sets of genes together. We did this systematically for all 29 cancer types, and found that accounting for replication strand bias has a negligible impact on both the identification of driver genes and the exome-wide dN/dS estimates. This may have been expected since replication strand asymmetry could only lead to noticeable biases in dN/dS if there were large systematic differences in sequence composition between genes on the leading strand and genes on the lagging strand. For example, bladder cancer has been highlighted as having one of the highest levels of replication strand asymmetry across TCGA datasets (Haradhvala et al., 2016). Calculating dN/dS separating genes according to replication strand or using all genes together (as done in our study) has no impact on the list of significant genes (the same six genes are found as significant) and has no detectable impact on the global dN/dS estimates. Global dN/dS values for missense mutations of 1.046 (CI95%: 1.009, 1.084) and 1.048 (CI95%: 1.022, 1.075), respectively, and for nonsense mutations of 1.191 (CI95%: 1.11, 1.278) and 1.191 (CI95%: 1.133, 1.252), respectively. Analogous results were obtained for *POLE* tumors, which display a high level of replication strand asymmetry.

#### Performance of different dN/dS models for driver discovery

Previous studies have highlighted the importance of adequately modeling the variation of mutation rates along the genome to identify driver (positively selected) genes with good specificity and sensitivity. Particularly, Lawrence et al. (2013) showed how models that do not account for the regional variation of the mutation rate along the genome can yield very long lists of false positives.

To evaluate the specificity of different methods in the presence of realistic levels of mutation rate variation along the genome, we can use realistic neutral simulations of somatic mutations. In line with ongoing international benchmarking efforts of driver discovery methods, we generated simulated neutral datasets by local randomization of somatic mutations from real whole-genome sequencing studies. Using data from 107 melanoma whole-genomes from ICGC, we first filtered out coding mutations from a panel of known driver genes, to minimize the presence of driver mutations, and then reassigned each mutation to a randomly selected position with an identical trinucleotide context within 50kb of its original position. This randomization procedure results on a neutral dataset that retains the same variation of mutation rates and mutational signatures across patients and across regions of the genome.

In a neutral dataset, robust methods for driver discovery with good specificity should not yield any significant hit. This can be formally evaluated by performing false discovery rate correction and by plotting the vector of *P*-values under the null model (neutral simulation) in a QQ-plot. The QQ-plot in Figure S2A reveals that the *dNdSunif* model yields a large number of false positives in the neutral simulation described above, as expected in the presence of large neutral variation of the mutation rate along the genome (Lawrence et al., 2013). In contrast, both *dNdSloc* (which estimates the local mutation rate from the synonymous substitutions in each gene) and *dNdScv* (which uses the regression framework in addition to local synonymous substitutions) have perfect specificity under the challenging conditions of the simulation above (Figure S2A). This result is representative of simulations performed under a variety of assumptions and starting datasets, even using simulated datasets with thousands of samples. The specificity of *dNdScv* has also been demonstrated by an international benchmarking exercise as part of the Pancancer Analysis of Whole-Genomes Consortium (PCAWG-ICGC) [*manuscript in preparation*].

Although both *dNdSloc* (which we used in Wong et al. [2014]) and *dNdScv* have good specificity under challenging conditions, they differ dramatically in terms of their sensitivity. This is shown in Figure S2B, which depicts the number of significant genes identified by both methods across the TCGA datasets analyzed in this study. While the *dNdSloc* model can detect a substantial number of positively selected genes in large datasets (Wong et al., 2014), *dNdScv* has higher sensitivity across datasets of any size, both when analyzing substitutions alone or when combining substitutions and indels. This is because *dNdScv* uses a joint likelihood function combining local information (synonymous mutations in a gene) and global information (negative binomial regression across genes) to model the background mutation rate of a gene, which leads to more confident (narrower) estimates. This is shown in Figures S2D–S2G with a series of examples of likelihood surfaces under *dNdSloc* and *dNdScv* for three different genes (two canonical driver genes, *PTEN* and *CDKN2A*, and one long passenger gene, *MUC16*) in two datasets of very different size (Lung-SCC and pancancer). *MUC16* is shown as an example of a challenging long passenger gene that has been reported as a common false positive in Lung-SCC and other datasets under simplistic models (Lawrence et al., 2013).

*dNdScv* has also been found to have similar or higher sensitivity than other driver discovery methods benchmarked in the Pancancer Analysis of Whole-Genomes Consortium (PCAWG-ICGC) [*manuscript in preparation*], including *MutSigCV* (Lawrence et al., 2014) and *oncodriveFML* (Mularoni et al., 2016).

#### Analyses of hypermutator tumors

##### Classification of hypermutator tumors by signature decomposition

Different analyses in this study excluded hypermutator tumors to ensure more accurate and representative results for the majority of tumor samples. For example, tumors with more than 500 substitutions/exome (comprising less than 8% of all samples in TCGA) were conservatively excluded for the analyses in Figures 3 and 4, and tumors with more than 3,000 coding substitutions (less than 1% of all samples in TCGA) were excluded for Figure 2.

To study in more depth the patterns of selection in hypermutator tumors (Figure 5), samples with more than 1,000 coding substitutions/exome (∼33 substitutions/Mb) were classified according to their dominant mutational process using mutational signature decomposition (Alexandrov et al., 2013). An expectation-maximization (EM) algorithm assuming a Poisson mixture model was used to calculate the relative contribution of 30 reference mutational signatures to each hypermutator sample (see below for details). Data of the 96-trinucleotide mutation probability for each signature was downloaded from the COSMIC database. Samples were classified according to their dominant mutational process when more than 50% of all of their mutations were attributed to a single mutational signature. For this classification, the two current mutational signatures attributed to *APOBEC* activity (signatures 2 and 13) were considered together. This conservatively classified 85% (246/288) of all hypermutator samples into five classes: *APOBEC* (COSMIC’s signatures 2 and/or 13), smoking (signature 4), mismatch-repair (MMR; signature 6), ultraviolet (UV; signature 7) and *POLE* (signature 10) (Figure 5B).

##### Expectation-Maximization algorithm for signature decomposition

Let ***m***_*i,j*_ be the 30x96 matrix of mutational signatures. For each sample, let ***c*** = (*c*_*1*_,*c*_*2*_,…,*c*_*30*_) be a vector representing the fraction of mutations in the sample attributed to each signature (with Σ_i_ *c*_*i*_ = 1). An EM algorithm is used to find the maximum-likelihood values of ***c*** iteratively. Step 1: the vector ***c*** is initialised with identical values for all elements (*c*_*i*_ = 1/30) or with random values. Step 2: given *c*_*i*_ and *m*_*i,j*_ the relative probability of a mutation (*h*) being caused by a particular signature in the sample is:

$$p_{h,i}=c_{i}\;m_{ij}/\Sigma_{k=\left(1,\ldots ,30\right)}\left(ck\;mk,j\right)$$

where *j* denotes the trinucleotide substitution type to which the mutation belongs. Step 3: summing *p*_*h,j*_ across all mutations gives an updated estimate of the contribution of each signature in the sample given the mutations observed in the sample.

$$c_{i}^{\prime }=\Sigma_{\text{h}}p_{h,i}$$

To obtain maximum likelihood estimates of *c*_*i*_, steps two and three are repeated iteratively until convergence of the *c*_*i*_ values (in particular, the iterative procedure was run until the sum of the absolute difference of the elements of ***c*** was < 10^−5^ between two successive iterations).

##### Neutral randomization with a 9-nt sequence context

In order to study the impact on dN/dS of the extended sequence-context dependence of *POLE* mutations, we generated a neutral dataset of mutations with an identical 9-nucleotide sequence-context (Figures 5C and 5D) by local randomization of observed mutations. Using TCGA whole-genome data from 505 tumors (Fredriksson et al., 2014) and signature decomposition, we identified five *POLE* hypermutator tumors. To avoid selection on coding sequences to have an impact on the neutral simulation, we first excluded all coding substitutions. We then randomly reallocated each non-coding mutation to a different position with an identical 9-nucleotide sequence context and within 1Mb of the original mutation. This generates a neutral dataset of random mutations that retains key original features of *POLE* mutations, including the 9-nucleotide mutational signature, the variation of mutation rates at megabase scale and the local strand biases. Running the trinucleotide dN/dS model on this simulated dataset yielded nearly identical dN/dS values to those observed in real *POLE* tumors from TCGA exomes (Figure 5C). This confirms that the relative excess of missense and particularly nonsense substitutions in *POLE* hypermutator tumors is not the result of selection for a large number of driver mutations but a consequence of the extended *POLE* mutational signature.

##### Conservative estimation of the density of driver mutations in known cancer genes

The analyses described in Figure 5, as well as those in Figure S1D,E, showed that mutational signatures extending beyond the trinucleotide context can lead to biases to dN/dS. Those biases appear negligible in most cohorts, as suggested by exome-wide dN/dS estimates very close and typically indistinguishable from 1 in likely-passenger genes or in hypermutator tumors (e.g., Figures 3G, 3H, and 5C). However, UV-induced mutations and, particularly, *POLE* mutations lead to significant biases to dN/dS under a trinucleotide substitution model (Figures 5C, 5D, S1D, and S1E).

In datasets significantly affected by mutational biases that are not captured by a trinucleotide model, the dN/dS ratios observed in likely-passenger genes can significantly deviate from 1 under neutrality (Figure 5C). Since selection in known cancer genes is typically much stronger than in likely-passenger genes, an approximate and slightly conservative way of removing mutational biases from dN/dS estimates in known cancer genes is to correct them by the dN/dS ratios observed in likely-passenger genes.

Original estimates of dN/dS in cancer genes, as shown in Figure 4A, were obtained using different ω parameters for the list of 369 cancer genes (Table S3) and for all other genes. This uses all genes to estimate the parameters of the substitution model (*r*_i_) more reliably. Using the notation described in STAR Methods:

$$n_{i,m,driver\_genes}∼Poisson\left(\lambda =t\;r_{i}L_{i,m}\omega_{m,driver\_genes}\right)$$

$$n_{i,m,other\_genes}∼Poisson\left(\lambda =t\;r_{i}L_{i,n}\omega_{m,other\_genes}\right)$$

In this framework, if we assume that ω parameters in likely-passenger genes are largely neutral and reflect mutational biases to dN/dS, we can obtain corrected estimates of dN/dS for known cancer genes (ω’) using:

$$n_{i,m,driver\_genes}∼Poisson\left(\lambda =t\;r_{i}L_{i,m}\omega_{m,driver\_genes}^{\prime }\omega_{m,likely\_passengers}\right)$$

$$n_{i,m,likely\_passengers}∼Poisson\left(\lambda =t\;r_{i}L_{i,n}\omega_{m,likely\_passengers}\right)$$

### Data and Software Availability

An R package with the code to run *dNdScv* has been made publicly available with this manuscript (https://github.com/im3sanger/dndscv).

## Author Contributions

I.M. developed the statistical methodology, analyzed, and interpreted the data. I.M., K.J.D., K.M.R., K.H., and P.V.L. downloaded the TCGA data and called somatic mutations. H.D. annotated driver mutations in breast cancer. M.G., M.R.S., and P.J.C. provided key advice. P.J.C. supervised the project. I.M. and P.J.C. wrote the manuscript.
