# Universal Patterns of Selection in Cancer and Somatic Tissues

**DOI:** 10.1016/j.cell.2018.06.001

**Published:** 2018-06-14

**Authors:** Iñigo Martincorena, Keiran M. Raine, Moritz Gerstung, Kevin J. Dawson, Kerstin Haase, Peter Van Loo, Helen Davies, Michael R. Stratton, Peter J. Campbell

(Cell *171*, 1029–1041.e1–e15; November 16, 2017)

It has come to our attention that in the Results and Discussion of the above article, we neglected to cite Davoli et al. (2013). This paper identified several of the cancer driver genes we mentioned in our paper and provided estimates of the number of genes under positive selection in cancer. The text and references in the online version of our paper have been corrected accordingly. We apologize for the omission and any inconvenience it may have caused to the scientific community.
