# Supplementary material for: Universal Patterns of Selection in Cancer and Somatic Tissues
Source: Cell. 2017 Nov 16;171(5):1029–1041.e21. doi: 10.1016/j.cell.2017.09.042 (PMC5720395; doi:10.1016/j.cell.2017.09.042)
Supplement: Document S1. Tables S1–S3 [file mmc1.pdf]

Cell, Volume *171*

Supplemental Information

Universal Patterns of Selection

in Cancer and Somatic Tissues

Iñigo Martincorena, Keiran M. Raine, Moritz Gerstung, Kevin J. Dawson, Kerstin Haase, Peter Van Loo, Helen Davies, Michael R. Stratton, and Peter J. Campbell

Table S1. [Related to Figure 1] List of cancer types and numbers of samples.

Cancer type	TCGA barcode	Short name used in this study	Samples (<3000 muts/exome)	Samples (<500 muts/exome)	TCGA calls used
Adrenocortical carcinoma	ACC	Adrenocortical	91	88	
Bladder Urothelial Carcinoma	BLCA	Bladder	390	337	
Breast invasive carcinoma	BRCA	Breast	702	689	
Cervical squamous cell carcinoma and endocervical adenocarcinoma	CESC	Cervix	38	36	
Colon and rectum adenocarcinoma	COAD+READ	Colorectum	266	226	
Esophageal carcinoma	ESCA	Esophagus	180	176	
Glioblastoma multiforme	GBM	Glioblastoma	255	255	
Head and Neck squamous cell carcinoma	HNSC	Head-neck	507	482	
Kidney Chromophobe	KICH	Kidney-ChRCC	66	65	Yes
Kidney renal clear cell carcinoma	KIRC	Kidney-RCC	314	314	
Kidney renal papillary cell carcinoma	KIRP	Kidney-Papill	275	275	
Acute Myeloid Leukemia	LAML	Myeloid-AML	136	136	Yes
Brain Lower Grade Glioma	LGG	CNS-LGG	467	466	
Liver hepatocellular carcinoma	LIHC	Liver-HCC	339	334	
Lung adenocarcinoma	LUAD	Lung-Adeno	367	299	
Lung squamous cell carcinoma	LUSC	Lung-SCC	167	152	
Mesothelioma	MESO	Mesothelioma	80	80	
Ovarian serous cystadenocarcinoma	OV	Ovary	262	262	
Pancreatic adenocarcinoma	PAAD	Pancreas-Adeno	145	145	Yes
Pheochromocytoma and Paraganglioma	PCPG	Pheo-Paragang	178	178	Yes
Prostate adenocarcinoma	PRAD	Prostate	497	494	Yes
Sarcoma	SARC	Sarcoma	247	241	
Skin Cutaneous Melanoma	SKCM	Melanoma	437	262	
Stomach adenocarcinoma	STAD	Stomach	360	291	
Testicular Germ Cell Tumors	TGCT	Testis	142	142	
Thyroid carcinoma	THCA	Thyroid	438	438	
Thymoma	THYM	Thymoma	29	28	
Uterine Corpus Endometrial Carcinoma	UCEC	Endometrium	233	180	
Uterine Carcinosarcoma	UCS	Uterus-CarSarc	56	55	

**All cancers with Caveman+Pindel calls**		**Pancancer***	**6642**	**6108**
**All cancers**		**Pancancer**	**7664**	**7126**

Table S2. [Related to Figure 2] Driver discovery by positive selection.

gene	CGCv73	Cancer5000-S	ACC	BLCA	BRCA	CESC	COREAD	ESCA	GBM	HNSC	KICH	KIRC	KIRP	LAML	LGG	LIHC	LUAD	LUSC	MESO	OV	PAAD	PCPG	PRAD	SARC	SKCM	STAD	TGCT	THCA	THYM	UCEC	UCS	PANCANCER	Combined
TP53	TRUE	TRUE	0	0	0	1	0	0	0	0	0	1	1	0	0	0	0	0	0	0	0	1	0	0	0	0	1	1	1	0	0	0	0
PTEN	TRUE	TRUE	1	4E-06	0	0.3067	0.0001	0.1709	0	0.0003	0.0042	5E-08	0.0001	1	0	0.6178	1	2E-08	1	1	1	1	3E-12	0.0023	0	1E-07	1	1	1	0	0	0	0
PIK3CA	TRUE	TRUE	1	0	0	0.0006	0	0.0001	0	0	1	1	1	1	0	1	0.7703	9E-05	1	1	1	1	0.6173	1	1	0	1	1	1	0	0	0	0
KRAS	TRUE	TRUE	1	0.0016	1	1	0	1	1	1	1	1	0.4375	1E-09	1	0.9195	0	1	1	0.5738	0	1	1	1	0.8176	0	0	0.1504	1	0	9E-08	0	0
ARID1A	TRUE	TRUE	1	0	0.0009	1	3E-05	0.0029	1	0.9505	1	1	1	1	1E-07	0.0004	6E-07	1	1	0.3728	0.0001	1	1	1	1	0	1	1	1	0	0.0079	0	0
RB1	TRUE	TRUE	1	0	1E-06	1	1	0.7945	0	0.0183	1	1	1	1	1	2E-05	2E-09	0.093	1	0.0025	1	1	1	0	5E-06	1	1	1	1	0.0357	0.4286	0	0
NF1	TRUE	TRUE	0.0116	0.2611	1	1	1	1	0	1	1	1	1	1	0	1	2E-06	0.8493	1	8E-06	1	0	1	1	0	1	1	1	1	1	1	0	0
FBXW7	TRUE	TRUE	1	2E-08	1	1	0	0.0004	1	1E-06	1	1	1	1	1	1	1	0.4524	1	1	1	1	1	1	1	0.0008	1	1	1	8E-10	0	0	0
NRAS	TRUE	TRUE	1	1	1	1	2E-09	1	1	1	1	1	1	0	0.3188	0.9977	1	1	1	1	1	1	1	1	0	1	7E-06	0	1	0.3135	1	0	0
PIK3R1	TRUE	TRUE	1	1	4E-05	1	0.4305	1	0	1	1	1	1	1	0	1	1	1	1	1	1	1	1	1	1	1	1	1	1	0	1E-05	0	0
CDKN2A	TRUE	TRUE	1	0	1	1	1	1	1	0	1	1	1	1	1	0.9195	0.9833	0.002	1	1	0	1	1	1	5E-12	1	1	1	1	1	1	0	0
NFE2L2	TRUE	TRUE	1	0.0045	1	0.2223	1	6E-08	1	2E-05	1	1	1	1	1	0.0008	0.852	6E-11	1	1	1	1	1	1	1	1	1	1	1	0.0007	1	0	0
SMAD4	TRUE	TRUE	1	1	0.7956	1	0	3E-07	1	0.2348	1	1	1	1	1	1	0.3242	1	1	1	0	1	1	1	1	6E-05	1	1	1	1	1	0	0
BAP1	TRUE	TRUE	1	0.2586	1	1	1	1	1	1	1	0	0.0003	1	1	5E-07	1	1	0	1	1	1	1	1	1	1	1	1	1	1	1	0	0
KMT2D	TRUE	TRUE	1	0	1	0.3067	1	0.1709	1	0	1	1	1	1	1	1	1	9E-05	1	1	1	1	0.0003	1	1	1	1	1	1	1	1	0	0
B2M	FALSE	TRUE	1	1	1	1	4E-09	1	1	0.001	1	1	1	1	1	1	1	0.0235	1	1	1	1	1	1	0.0001	1E-10	1	1	1	1	1	0	0
KEAP1	FALSE	TRUE	1	1	1	1	1	1	1	0.0003	1	1	1	1	1	3E-06	0	2E-06	1	1	1	1	1	1	1	1	1	1	1	1	1	1.54E-10	0
BRAF	TRUE	TRUE	1	1	1	1	1E-07	1	0.6176	1	1	1	1	1	1	1	0.0552	1	1	1	1	1	1	1	0	1	1	0	1	1	1	0	0
ARID2	TRUE	TRUE	1	0.0012	1	1	1	1	1	0.0136	1	1	1	1	0.0998	8E-06	0.2048	1	1	1	1	1	1	1	0	0.0138	1	1	1	1	1	0	0
CTNNB1	TRUE	TRUE	9E-09	1	1	1	1	1	1	1	1	1	1	1	1	0	0.4782	1	1	1	1	1	0.1016	1	1	1	1	1	1	0	1	0	0
IDH1	TRUE	TRUE	1	1	1	1	1	1	4E-10	1	1	1	1	0	0	1	1	1	1	1	1	1	1	1	0.98	1	1	1	1	1	1	0	0
HLA-B	FALSE	TRUE	1	1	1	1	0.0005	0.517	1	0	1	1	1	1	0.6239	0.0153	1	1	1	1	1	1	1	1	1	3E-09	1	1	1	1	1	0	0
EGFR	TRUE	TRUE	1	1	1	1	1	1	0	1	1	1	1	1	0	1	1E-07	1	1	1	1	1	1	1	1	1	1	1	1	1	1	3.05E-06	0
ATRX	TRUE	FALSE	1	1	1	1	1	1	8E-05	1	1	1	1	1	0	1	1	1	1	1	1	0.2481	1	0	1	1	1	1	1	1	1	0	0
HRAS	TRUE	TRUE	1	3E-05	1	1	1	1	1	0	1	1	1	1	1	1	1	1	1	1	1	0	1	1	0.9412	1	1	1	0.6459	1	1	0	0
FOXA1	TRUE	TRUE	1	9E-05	2E-11	1	1	1	1	1	1	1	1	1	1	1	1	1	1	1	1	1	0	1	1	1	1	1	1	1	1	1.47E-07	0
KDM6A	TRUE	TRUE	1	0	1	1	1	1	1	0.0063	1	1	0.0001	1	1	1	1	0.1804	1	1	1	1	0.0076	1	1	1	1	1	1	1	1	0	0
NOTCH1	TRUE	TRUE	1	1	1	1	1	0.0479	1	0	1	1	1	1	3E-12	1	1	0.0348	1	1	1	1	1	1	1	1	1	1	1	1	1	1.88E-11	0
FAT1	FALSE	TRUE	1	0.0002	1	1	1	1	1	0	1	1	1	1	1	1	1	0.0904	1	1	1	1	1	1	1	1	1	1	1	0.0115	1	0	0
KMT2C	TRUE	TRUE	1	1E-05	2E-11	0.5303	1	1	1	1	1	1	0.6858	1	1	1	1	0.6958	1	1	1	1	0.0729	1	1	0.9439	1	1	1	1	1	0	0
RUNX1	TRUE	TRUE	1	0.279	0	1	1	1	1	1	1	1	1	0	1	1	1	1	1	1	1	1	1	1	1	1	1	1	1	1	1	3.34E-07	0
SETD2	TRUE	TRUE	1	1	1	1	1	1	1	1	1	0	0.0136	1	1	1	0.0295	1	0.0027	1	1	1	1	1	1	1	1	1	1	1	1	0	0
APC	TRUE	TRUE	1	1	1	1	0	1	1	1	1	1	1	1	1	1	1	1	1	1	1	1	0.027	1	1	9E-05	1	1	1	1	1	0	0
SPOP	TRUE	TRUE	1	1	1	1	1	1	1	1	1	1	1	1	1	1	1	1	1	1	1	1	0	1	1	1	1	1	1	7E-06	0.3904	1.71E-08	0
RHOA	TRUE	TRUE	1	6E-07	0.765	1	1	1	1	0.227	1	1	1	1	1	1	1	1	1	1	1	1	1	1	1	3E-06	1	1	1	1	1	1.68E-06	1.31E-06
STAG2	TRUE	TRUE	1	0	1	1	1	1	0.0354	1	1	1	0.0322	0.0083	1	1	1	1	1	1	1	1	1	1	1	1	1	1	1	1	1	2.29E-13	0
NF2	TRUE	FALSE	1	1	1	1	1	1	1	0.6342	1	1	3E-05	1	1	1	1	1	0	1	1	1	1	1	1	1	1	1	1	1	1	0	0
CDKN1B	TRUE	TRUE	1	1	5E-08	1	1	1	1	1	1	1	1	1	1	1	0.304	1	1	1	1	1	0.0001	1	1	1	1	1	1	1	1	1.02E-10	7.47E-10
RNF43	TRUE	FALSE	1	1	1	1	0.0406	1	1	1	1	1	1	1	1	1	1	1	1	1	0.005	1	0.9473	1	1	0.0004	1	1	1	0.0007	1	7.48E-09	5.61E-08
HLA-A	TRUE	TRUE	1	1	1	1	0.0154	1	1	6E-13	1	1	1	1	1	1	1	0.2403	1	1	1	1	1	1	1	0.0144	1	1	1	1	1	3.77E-12	1.20E-12
CTCF	FALSE	TRUE	1	1	0.0045	1	0.5419	1	1	0.042	1	1	1	1	1	1	1	1	1	1	1	1	1	1	1	1	1	1	1	0	1	9.75E-12	0
ZNF750	FALSE	TRUE	1	0.2134	1	1	1	8E-05	1	1E-07	1	1	1	1	1	1	1	1	1	1	1	1	1	1	1	1	1	1	1	1	1	6.57E-06	2.19E-07
RBM10	FALSE	TRUE	1	0.0008	1	1	1	1	1	1	1	1	1	1	1	1	8E-10	1	1	1	1	1	1	1	1	1	1	1	1	1	1	3.16E-12	2.22E-11
EP300	TRUE	TRUE	1	2E-10	1	1	1	1	1	0.0014	1	1	1	1	1	1	1	1	1	1	1	1	1	1	1	1	1	1	1	1	1	3.17E-09	4.20E-10
CASP8	TRUE	TRUE	1	1	0.1734	1	1	1	1	0	1	1	1	1	1	1	1	1	1	1	1	1	1	1	1	0.031	1	1	1	0.3595	1	0	0
ZFP36L1	FALSE	FALSE	1	0	0.0035	1	1	1	1	1	1	1	1	1	1	1	1	1	1	1	1	1	1	1	1	1	1	1	1	1	1	2.95E-11	0
PBRM1	TRUE	TRUE	1	1	1	1	1	1	1	1	1	0	0.0078	1	1	1	1	1	1	1	0.6456	1	1	1	0.98	1	1	1	1	1	1	0	0
RAC1	TRUE	TRUE	1	1	1	1	1	1	1	0.001	1	1	1	1	1	1	1	1	1	1	1	1	1	1	4E-07	1	1	1	1	1	1	5.34E-06	3.63E-07
PPP2R1A	TRUE	TRUE	1	1	1	1	1	1	1	1	1	1	1	1	1	1	1	1	1	1	1	1	1	1	1	1	1	1	1	3E-05	0	0.00078545	0
CDH1	TRUE	TRUE	1	1	0	1	1	1	1	1	1	1	1	1	1	1	1	1	1	1	1	1	1	1	1	0.0574	1	1	1	1	1	1.17E-13	0
KIT	TRUE	TRUE	1	1	1	1	1	1	1	1	1	1	1	7E-09	1	1	1	1	1	1	1	1	1	1	1	1	0	1	1	1	1	0.07344232	0
IDH2	TRUE	TRUE	1	1	1	1	1	1	1	1	1	1	1	0	0	1	1	1	1	1	1	1	1	1	1	1	1	1	1	1	1	0.10281876	0
ARHGAP35	FALSE	TRUE	1	0.073	0.9063	1	1	1	1	1	1	1	1	1	1	1	1	1	1	1	1	1	1	1	1	1	1	1	1	1E-06	1	1.23E-12	8.51E-12
MAP3K1	TRUE	TRUE	1	1	0	1	1	1	1	1	1	1	1	1	1	1	1	1	1	1	1	1	1	1	1	1	1	1	1	0.1551	1	0	0
ELF3	FALSE	TRUE	1	0	1	1	0.8372	1	1	1	1	1	1	1	1	1	1	1	1	1	1	1	1	1	1	0.3507	1	1	1	1	1	0	0
SMARCA4	TRUE	TRUE	1	1	1	1	1	1	1	1	1	1	1	1	4E-06	1	0.0006	1	1	1	1	1	1	1	1	1	1	1	1	1	1	1.42E-05	7.54E-06
FUBP1	TRUE	FALSE	1	1	1	1	1	1	1	1	1	1	1	1	0	1	1	0.5925	1	1	1	1	1	1	1	1	1	1	1	1	1	1.19E-08	0
SOX9	FALSE	FALSE	1	1	1	1	0	1	1	1	1	1	1	1	1	1	1	1	1	1	1	1	1	1	1	0.7886	1	1	1	1	1	5.48E-09	0
CDKN1A	FALSE	TRUE	1	0	1	1	1	1	1	1	1	1	1	1	1	1	1	1	1	1	1	1	1	1	1	1	1	1	1	1	1	0	0
STK11	TRUE	TRUE	1	1	1	1	1	1	1	1	1	1	1	1	1	1	0	1	1	1	1	1	1	1	1	1	1	1	1	1	1	0	0
VHL	TRUE	TRUE	1	1	1	1	1	1	1	1	1	0	1	1	1	1	1	1	1	1	1	1	1	1	1	1	1	1	1	1	1	0	0
GATA3	TRUE	TRUE	1	1	0	1	1	1	1	1	1	1	1	1	1	1	1	1	1	1	1	1	1	1	1	1	1	1	1	1	1	0	0
AJUBA	FALSE	TRUE	1	1	1	1	1	1	1	0	1	1	1	1	1	1	1	1	1	1	1	1	1	1	1	1	1	1	1	1	1	1.26E-10	0
CIC	TRUE	FALSE	1	1	1	1	1	1	1	1	1	1	1	1	0	1	1	1	1	1	1	1	1	1	1	1	1	1	1	1	1	1.46E-06	0
CREBBP	TRUE	TRUE	1	6E-08	1	1	1	1	1	1	1	1	1	1	1	1	1	1	1	1	1	1	1	1	1	1	1	1	1	1	1	1.09E-06	1.35E-07
NSD1	TRUE	TRUE	1	1	1	1	1	1	1	0	1	1	1	1	1	1	1	1	1	1	1	1	1	1	1	1	1	1	1	1	1	2.06E-06	0
MAP2K4	TRUE	TRUE	1	1	0	1	0.8372	1	1	1	1	1	1	1	1	1	1	1	1	1	1	1	1	1	1	1	1	1	1	1	1	2.72E-06	0
TGFBR2	FALSE	TRUE	1	1	1	1	1	0.1709	1	1E-07	1	1	1	1	1	1	1	1	1	1	0.0882	1	1	1	1	1	1	1	1	1	1	0.00019263	2.11E-07
AXIN1	TRUE	FALSE	1	1	1	1	1	1	1	1	1	1	1	1	1	0	1	1	1	1	1	1	1	1	1	1	1	1	1	1	1	5.43E-06	0
CBFB	TRUE	TRUE	1	1	0	1	1	1	1	1	1	1	1	0.0213	1	1	1	1	1	1	1	1	1	1	1	1	1	1	1	1	1	0.00044676	0
TSC1	TRUE	TRUE	1	2E-13	1	1	1	1	1	1	1	1	1	1	1	1	1	1	1	1	1	1	1	1	1	1	1	1	1	1	1	3.25E-05	4.06E-13
ZFP36L2	FALSE	FALSE	1	1	1	1	4E-05	1	1	0.213	1	1	1	1	1	1	1	1	1	1	1	1	1	1	1	0.294	1	1	1	0.2778	1	3.82E-06	2.88E-05
RASA1	FALSE	TRUE	1	1	1	1	1	1	1	0.0035	1	1	1	1	1	1	1	0.1046	1	1	1	1	1	1	1	0.8147	1	1	1	0.3446	1	4.67E-09	3.45E-08
FGFR3	TRUE	TRUE	1	0	1	1	1	1	1	0.1934	1	1	1	1	1	1	1	1	1	1	1	1	1	1	1	1	1	1	1	1	1	0.00136759	0
EPHA2	FALSE	TRUE	1	0.3116	1	1	1	1	1	4E-08	1	1	1	1	1	1	1	1	1	1	1	1	1	1	1	1	1	1	1	1	1	0.00078764	7.37E-08

FGFR2	TRUE	TRUE	1	1	1	1	1	1	1	1	1	1	1	1	1	1	1	1	1	1	1	1	1	1	1	1	1	1	1	1E-07	1	0.00058507	2.13E-07
DNMT3A	TRUE	TRUE	1	1	1	1	1	1	1	1	1	1	1	0	0.0424	1	1	1	1	1	1	1	1	1	1	1	1	1	1	1	1	0.02803229	0
KANSL1	FALSE	FALSE	1	0.0211	1	1	1	1	1	0.0611	1	1	1	1	1	1	1	1	1	1	1	1	1	1	1	1	1	1	1	1	1	8.45E-09	6.43E-08
TCF12	TRUE	FALSE	1	1	1	1	1	1	0.0473	1	1	1	1	1	3E-08	1	1	1	1	1	1	1	1	1	1	1	1	1	1	1	1	0.04406101	6.70E-08
ATM	TRUE	TRUE	1	0.0662	1	1	0.6532	1	1	1	1	0.4802	1	1	1	1	0.304	1	1	1	1	1	0.2595	1	1	1	1	1	1	0.3595	1	4.01E-07	2.85E-06
ALB	FALSE	FALSE	1	1	1	1	1	1	1	1	1	1	1	1	1	0	1	1	1	1	1	1	1	1	1	1	1	1	1	1	1	0.00604243	0
MEN1	TRUE	FALSE	3E-08	1	1	1	1	1	1	1	1	1	1	1	1	1	1	1	1	1	1	1	1	1	1	1	1	1	1	1	1	0.00615463	1.16E-08
ERBB2	TRUE	TRUE	1	0.0003	0.0915	1	1	0.2701	1	1	1	1	1	1	1	1	1	1	1	1	1	1	1	1	1	1	1	1	1	1	1	1.18E-05	8.73E-05
PSIP1	TRUE	FALSE	1	5E-05	1	1	1	1	1	0.5246	1	1	1	1	1	1	1	1	1	1	1	1	1	1	1	1	1	1	1	1	1	6.57E-06	5.03E-05
MGA	FALSE	TRUE	1	1	1	1	1	1	1	1	1	1	1	1	1	1	0.0007	1	1	1	1	1	1	1	1	1	1	1	1	0.1552	1	3.41E-06	2.54E-05
AMER1	TRUE	FALSE	1	1	1	1	0	1	1	1	1	1	1	1	1	1	1	1	1	1	1	1	1	1	1	1	1	1	1	1	1	0.01417868	0
FLT3	TRUE	TRUE	1	1	1	1	1	1	1	1	1	1	1	0	1	1	1	1	1	1	1	1	1	1	1	1	1	1	1	1	1	0.04614891	0
KMT2B	FALSE	TRUE	1	1	1	1	0.6532	1	1	0.0209	1	1	1	1	1	0.6779	1	1	1	1	1	1	1	1	1	0.5518	1	1	1	0.2398	1	2.24E-06	1.60E-05
ZFHX3	FALSE	TRUE	1	1	0.992	1	1	1	1	1	1	1	1	1	1	1	1	1	1	1	1	1	0.0206	1	1	1	1	1	1	5E-06	1	0.08086603	8.02E-06
ARID1B	TRUE	FALSE	1	1	1	1	1	1	1	1	1	1	1	1	1	1	1	1	1	1	1	1	1	1	1	0.4489	1	1	1	0.212	1	1.39E-09	1.02E-08
BMPR2	FALSE	FALSE	1	1	1	1	1E-04	1	1	1	1	1	1	1	1	1	1	1	1	1	1	1	1	1	1	0.031	1	1	1	1	1	0.00027575	0.00014822
TET2	TRUE	TRUE	1	1	1	1	1	1	1	1	1	1	1	9E-13	1	1	1	1	1	1	1	1	1	1	1	1	1	1	1	1	1	0.1515449	1.58E-12
PTPN11	TRUE	TRUE	1	1	1	1	1	1	1	1	1	1	1	3E-08	0.1445	1	1	1	1	1	1	1	1	1	1	1	1	1	1	1	1	1	5.53E-08
MAP2K7	FALSE	FALSE	1	1	1	1	1	1	1	1	1	1	1	1	1	1	1	1	1	1	1	1	1	1	1	3E-07	1	1	1	1	1	0.10263669	2.74E-07
NCOR1	TRUE	TRUE	1	0.4072	2E-06	1	1	1	1	1	1	1	1	1	1	1	1	1	1	1	1	1	1	1	1	1	1	1	1	1	1	0.03086232	3.82E-06
GTF2I	FALSE	FALSE	1	0.23	1	1	1	1	1	1	1	1	1	1	1	1	1	1	1	1	1	1	1	1	1	1	1	1	0	1	1	0.90007798	0
TBX3	TRUE	TRUE	1	1	1E-09	1	1	1	1	1	1	1	1	1	1	1	1	1	1	1	1	1	1	1	1	1	1	1	1	1	1	0.2182296	1.87E-09
ARID5B	FALSE	TRUE	1	1	1	1	1	1	1	1	1	1	1	1	1	1	1	1	1	1	1	1	1	1	1	1	1	1	1	2E-08	1	0.24945188	2.50E-08
RHOB	FALSE	FALSE	1	2E-10	1	1	1	1	1	1	1	1	1	1	1	1	1	1	1	1	1	1	1	1	1	1	1	1	1	1	1	0.26519362	5.26E-10
CEBPA	TRUE	TRUE	1	1	1	1	1	1	1	1	1	1	1	0	1	1	1	1	1	1	1	1	1	1	1	1	1	1	1	1	1	0.29083518	0
BCOR	TRUE	TRUE	1	1	1	1	1	1	1	1	1	1	1	1	0.0139	1	1	1	1	1	1	1	1	1	1	9E-05	1	1	1	1	1	0.00224075	0.00010611
NPM1	TRUE	TRUE	1	1	1	1	1	1	1	1	1	1	1	0	1	1	1	1	1	1	1	1	1	1	1	1	1	1	1	1	1	0.46243413	0
ACVR2A	FALSE	FALSE	1	1	1	1	0.6169	1	1	1	1	1	1	1	1	0.0001	1	1	1	1	1	1	1	1	1	0.7953	1	1	1	1	1	9.40E-05	0.00011753
KDM5C	TRUE	TRUE	1	1	1	1	1	1	1	1	1	2E-08	1	1	1	1	1	1	1	1	1	1	1	1	1	1	1	1	1	1	1	0.78923297	1.57E-08
ZBTB20	FALSE	FALSE	1	1	1	1	1	1	1	1	1	1	1	1	2E-10	1	1	1	1	1	1	1	1	1	1	1	1	1	1	1	1	0.88816717	4.29E-10
RPL22	TRUE	FALSE	1	1	1	1	1	1	1	1	1	1	1	1	1	0.1191	1	1	1	1	0.3703	1	1	1	1	0.4669	1	1	1	0.955	0.804	3.41E-06	2.51E-05
ERCC2	TRUE	TRUE	1	6E-07	1	1	1	1	1	1	1	1	1	1	1	1	1	1	1	1	1	1	1	1	1	1	1	1	1	1	1	0.5618129	1.43E-06
WT1	TRUE	TRUE	1	1	1	1	1	1	1	1	1	1	1	5E-07	1	1	1	1	1	1	1	1	1	1	1	1	1	1	1	1	1	0.87331724	9.06E-07
MET	TRUE	TRUE	1	1	1	1	1	1	1	1	1	1	1E-06	1	1	1	1	1	1	1	1	1	1	1	1	1	1	1	1	1	1	1	1.80E-07
HIST1H3B	TRUE	TRUE	1	0.024	0.0007	1	1	1	1	1	1	1	1	1	1	1	1	1	1	1	1	1	1	1	1	1	1	1	1	1	1	0.00067255	0.00113845
TOP2A	FALSE	FALSE	1	1	1	1	1	1	1	1	1	1	1	1	1	1	1	1	1	6E-06	1	1	1	1	1	1	1	1	1	1	1	0.83849076	1.59E-06
U2AF1	TRUE	TRUE	1	1	1	1	1	1	1	1	1	1	1	4E-06	1	1	0.9471	1	1	1	1	1	1	1	1	1	1	1	1	1	1	0.83801773	6.53E-06
HLA-C	FALSE	FALSE	1	1	1	1	0.0001	1	1	0.5246	1	1	1	1	1	1	1	1	1	1	1	1	1	1	0.98	0.1365	1	1	1	1	1	0.01941588	0.00019099
NIPBL	FALSE	FALSE	1	1	1	1	1	1	1	1	1	1	1	1	6E-06	1	1	1	1	1	1	1	1	1	1	1	1	1	1	1	1	0.30945572	1.22E-05
CD58	FALSE	FALSE	1	1	1	1	0.0001	1	1	1	1	1	1	1	1	1	1	1	1	1	1	1	1	1	1	1	1	1	1	1	1	0.00238168	0.00016989
DDX3X	FALSE	TRUE	1	1	1	1	1	1	1	0.4274	1	1	1	1	1	1	1	1	1	1	1	1	1	1	0.0001	1	1	1	1	1	1	0.00615463	0.00013047
AKT1	TRUE	TRUE	1	1	8E-06	1	1	1	1	1	1	1	1	1	1	1	1	1	1	1	1	1	1	1	1	1	1	0.9906	1	1	1	0.6595108	1.37E-05
PRKAR1A	TRUE	FALSE	4E-05	1	1	1	1	1	1	1	1	1	1	1	1	1	1	1	1	1	1	1	1	1	1	1	1	1	1	1	1	0.50703231	1.64E-05
CUL3	FALSE	FALSE	1	1	1	1	1	1	1	0.594	1	1	0.0004	1	1	1	1	0.0904	1	1	1	1	1	1	1	1	1	1	1	1	1	0.08420611	0.00023555
PHF6	TRUE	TRUE	1	1	1	1	1	1	1	1	1	1	1	2E-05	1	1	1	1	1	1	1	1	1	1	1	1	1	1	1	1	1	0.9187309	3.80E-05
KMT2A	TRUE	TRUE	1	0.0007	1	1	1	1	1	1	1	1	1	1	1	1	1	1	1	1	1	1	1	1	0.9959	1	1	1	1	1	1	0.0011103	0.00172423
SPTAN1	FALSE	FALSE	1	4E-05	1	1	1	1	1	1	1	1	1	1	1	1	1	1	1	1	1	1	1	1	1	1	1	1	1	1	1	0.90007798	8.14E-05
TCF7L2	TRUE	TRUE	1	0.9119	1	1	7E-05	1	1	1	1	1	1	1	1	1	1	1	1	1	1	1	1	1	1	1	1	1	1	1	1	0.49525806	9.51E-05
MUC6	FALSE	FALSE	1	1	1	1	1	0.0552	1	1	1	1	1	1	1	1	1	1	1	1	1	1	1	1	1	0.0004	1	1	1	1	1	0.33001607	0.00046044
BRCA1	TRUE	TRUE	1	1	0.934	1	1	1	1	1	1	1	1	1	1	1	1	1	1	0.0002	1	1	1	1	1	1	1	1	1	1	1	0.23310704	8.23E-05
TGIF1	FALSE	FALSE	1	1	1	1	1E-04	1	1	1	1	1	1	1	1	1	1	1	1	1	1	1	1	1	1	1	1	1	1	1	1	0.34894575	0.00014236
PPM1D	FALSE	TRUE	1	1	1	1	1	1	1	0.1604	1	1	1	1	1	1	1	1	1	1	1	1	1	1	1	1	1	0.1184	1	1	1	0.00024317	0.0016326
FOXQ1	FALSE	TRUE	1	0.0001	1	1	1	1	1	1	1	1	1	1	1	1	1	1	1	1	1	1	1	1	1	1	1	1	1	1	1	0.2182296	0.00030063
LATS2	FALSE	FALSE	1	1	1	1	1	1	1	1	1	1	1	1	1	1	1	1	0.0002	1	1	1	1	1	1	1	1	1	1	1	1	0.9187309	8.98E-05
ERBB3	TRUE	TRUE	1	0.0071	1	1	1	1	1	1	1	1	1	1	1	1	1	1	1	1	1	1	1	1	1	0.0477	1	1	1	1	1	0.00343843	0.0184373
EIF1AX	FALSE	FALSE	1	1	1	1	1	1	1	1	1	1	1	1	1	1	1	1	1	1	1	1	1	1	1	1	1	0.0005	1	1	1	0.29543714	0.00014495
CDK12	TRUE	TRUE	1	1	1	1	1	1	1	1	1	1	1	1	1	1	1	1	1	0.018	1	1	0.0076	1	1	1	1	1	1	1	1	0.03438913	0.00476591
EEF1A1	FALSE	FALSE	1	0.4849	1	1	1	1	0.2286	1	1	1	1	1	1	0.1191	1	1	1	1	1	1	1	1	1	1	1	1	1	1	1	0.00063068	0.00429545
SMC3	FALSE	TRUE	1	1	1	1	1	1	1	1	1	1	1	0.0003	1	1	1	1	1	1	1	1	1	1	1	1	1	1	1	1	1	0.830133	0.00040765
RXRA	FALSE	TRUE	1	0.0002	1	1	1	1	1	1	1	1	1	1	1	1	1	1	1	1	1	1	1	1	1	1	1	1	1	1	1	0.96604448	0.00052741
ZNRF3	FALSE	FALSE	1	1	1	1	0.0998	1	1	1	1	1	1	1	1	1	1	1	1	1	1	1	1	1	1	0.4669	1	1	1	1	1	0.00064519	0.00441083
ASXL1	TRUE	TRUE	1	0.0544	1	1	1	1	1	0.0252	1	1	1	0.0556	1	1	1	1	1	1	1	1	1	1	1	1	1	1	1	1	1	0.04618088	0.04801508
GPS2	FALSE	TRUE	1	1	0.001	1	1	1	1	1	1	1	1	1	1	1	1	1	1	1	1	1	1	1	1	1	1	1	1	1	1	0.17401581	0.0016326
MTOR	FALSE	TRUE	1	1	1	1	1	1	1	1	1	0.0034	1	1	1	1	1	1	1	1	1	1	1	1	1	1	1	1	1	1	1	0.04557862	0.00196559
FOXA2	FALSE	FALSE	1	1	1	1	1	1	1	1	1	1	1	1	1	1	1	1	1	1	1	1	1	1	1	1	1	1	1	0.0008	1	0.58578057	0.00124329
ZBTB7B	FALSE	FALSE	1	1	1	1	1	1	1	1	1	1	1	1	1	1	1	1	1	1	1	1	1	1	1	1	1	1	1	1	0.001	0.88637625	0.00074269
KLF5	FALSE	FALSE	1	0.0009	1	1	1	1	1	1	1	1	1	1	1	1	1	1	1	1	1	1	1	1	1	1	1	1	1	1	1	0.33688809	0.00247316
BRD7	FALSE	FALSE	1	1	1	1	1	1	1	1	1	1	1	1	1	0.0814	1	1	1	1	1	1	1	1	0.0409	1	1	1	1	1	1	0.00671581	0.04095633
RACGAP1	FALSE	FALSE	1	1	1	1	1	1	1	1	1	1	1	1	1	1	1	1	1	1	1	1	1	1	1	1	0.0017	1	1	1	1	0.96604448	0.00062196
CHD4	FALSE	TRUE	1	1	1	1	1	1	1	1	1	1	1	1	1	1	1	1	1	1	1	1	1	1	1	1	1	1	1	0.0373	0.0084	0.64660474	0.00653055
OR4N2	FALSE	FALSE	1	0.7757	1	1	1	1	1	1	1	1	1	1	1	1	1	1	1	1	1	1	1	1	0.3443	1	1	1	1	1	1	0.00086898	0.0060264
RPS6KA3	FALSE	FALSE	1	1	1	1	1	1	1	1	1	1	1	1	1	0.0014	1	1	1	1	1	1	1	1	1	1	1	1	1	1	1	0.94737351	0.00141305
CUX1	TRUE	TRUE	1	1	1	1	1	1	1	1	1	1	1	1	1	1	1	1	1	1	1	1	1	1	1	1	1	1	1	1	1	0.00055579	0.00371533
FOSL2	FALSE	FALSE	1	0.638	1	1	1	1	1	0.0413	1	1	1	1	1	1	1	1	1	1	1	1	1	1	1	1	1	1	1	1	1	0.00429977	0.0292344
TG	FALSE	FALSE	1	1	1	1	1	1	1	1	1	1	1	1	1	1	1	1	1	1	1	1	1	1	1	1	1	0.0051	1	1	1	0.44669268	0.00170881
PDYN	FALSE	FALSE	1	1	1	1	1	1	1	1	1	1	1	1	1	1	1	0.0572	1	1	1	1	1	1	1	1	1	1	1	1	1	0.00341608	0.02299129
ZFX	FALSE	FALSE	1	1	1	1	1	1	1	1	1	1	1	1	1	1	1	1	1	1	1	1	1	1	0.0037	1	1	1	1	1	1	0.63782246	0.00367305
TSC2	TRUE	FALSE	1	1	1	1	1	1	1	1	1	1	1	1	1	0.0044	1	1	1	1	1	1	1	1	1	1	1	1	1	1	1	0.44363526	0.00454191

HIST1H1C	FALSE	FALSE	1	1	1	1	1	1	1	1	1	1	1	1	1	0.0893	1	1	1	1	1	1	1	1	1	1	1	1	1	1	1	0.00421943	0.02854706
SMTNL2	FALSE	FALSE	1	1	1	1	1	1	1	1	1	1	1	1	1	1	1	1	1	1	1	1	1	1	1	1	1	1	1	0.0031	1	0.99831572	0.00461224
ZNF800	FALSE	FALSE	1	1	1	1	1	1	1	1	1	0.0768	1	1	1	1	0.2713	1	1	1	1	1	1	1	1	1	1	1	1	1	1	0.01941588	0.04095633
CSDE1	FALSE	FALSE	1	1	1	1	1	1	1	1	1	1	1	1	1	1	1	1	1	1	1	0.0186	1	1	1	1	1	1	1	1	1	0.25806912	0.0044817
HOXB3	FALSE	FALSE	1	1	1	1	1	1	1	1	1	1	1	1	1	1	0.014	0.3637	1	1	1	1	1	1	1	1	1	1	1	1	1	0.33628085	0.01266151
CDH10	FALSE	FALSE	1	1	1	1	0.4305	1	1	0.5646	1	1	1	1	1	1	0.9471	0.5194	1	1	1	1	1	1	1	1	1	1	1	1	1	0.00604243	0.0401477
CMTR2	FALSE	FALSE	1	1	1	1	1	1	1	1	1	1	1	1	1	1	0.0324	1	1	1	1	1	1	1	1	1	1	1	1	1	1	0.04038863	0.03135695
GAGE12J	FALSE	FALSE	1	1	0.009	1	1	1	1	1	1	1	1	1	1	1	1	1	1	1	1	1	1	1	1	1	1	1	1	1	1	0.29543714	0.01545926
CIB3	FALSE	FALSE	1	1	0.7078	1	1	1	1	1	1	1	1	1	1	1	1	1	1	0.028	1	1	1	1	1	0.5338	1	1	1	1	1	0.27941732	0.01467005
LZTR1	FALSE	FALSE	1	1	1	1	1	1	0.0193	1	1	1	1	1	1	1	1	1	1	1	1	1	1	1	1	1	1	1	1	1	1	0.24945188	0.01447673
MBD6	FALSE	FALSE	1	1	0.5823	1	0.2619	1	1	0.711	1	1	1	1	1	1	1	1	1	1	1	1	1	1	1	0.3531	1	1	1	0.955	1	0.02111719	0.13103077
RPL5	TRUE	TRUE	1	1	1	1	1	1	0.0342	1	1	1	1	1	1	1	1	1	1	1	1	1	1	1	0.907	1	1	1	1	1	1	0.1239127	0.02671915
TYRO3	FALSE	FALSE	1	1	1	1	1	1	1	1	1	1	1	1	1	1	1	1	1	1	0.0151	1	1	1	1	1	1	1	1	1	1	0.99014686	0.00810402
KRT5	FALSE	FALSE	1	1	1	1	1	1	1	0.0097	1	1	1	1	1	1	1	1	1	1	1	1	1	1	1	1	1	1	1	1	1	0.82558466	0.0178078
JAK1	TRUE	FALSE	1	1	1	1	1	1	1	1	1	1	1	1	1	1	1	1	1	1	1	1	1	1	1	1	1	1	1	0.0262	1	0.13639683	0.04034633
HIST1H2BD	FALSE	FALSE	1	1	1	1	1	1	1	1	1	1	1	1	1	1	1	1	1	1	1	1	1	1	1	1	1	1	1	0.0118	1	0.88319886	0.01810247
ATP1B1	FALSE	FALSE	1	1	1	1	1	1	1	1	1	1	0.0481	1	1	1	1	1	1	1	1	1	1	1	1	1	1	1	1	1	1	0.13639683	0.03306732
OR11H1	FALSE	FALSE	1	1	1	1	1	1	1	0.6342	1	1	1	1	1	1	1	1	1	1	1	1	1	1	1	0.9425	1	1	1	1	1	0.00773883	0.05209799
ASXL2	FALSE	FALSE	1	0.0415	1	1	1	1	1	1	1	1	1	1	1	1	1	1	1	1	1	1	1	1	1	1	1	1	1	1	1	0.06282406	0.10127399
UPF3A	FALSE	FALSE	1	1	1	1	0.6467	1	1	1	1	1	1	1	1	1	1	1	1	1	1	1	1	1	1	1	1	1	1	0.029	1	0.34119323	0.04573829
PPP6C	TRUE	TRUE	1	1	1	1	1	1	1	1	1	1	1	1	1	1	1	1	1	1	1	1	1	1	0.0202	1	1	1	1	1	1	0.73382397	0.02043697
EPS8	FALSE	FALSE	1	0.0114	1	1	1	1	1	1	1	1	1	1	1	1	1	1	1	1	1	1	1	1	1	1	1	1	1	1	1	0.91929348	0.02940189
HBD	FALSE	FALSE	1	1	1	1	1	1	1	1	1	1	1	1	1	1	1	1	1	1	1	1	1	1	0.0504	1	1	1	1	1	1	0.11641455	0.05237895
PSG4	FALSE	FALSE	1	1	1	1	1	1	1	1	1	1	1	1	1	1	1	1	1	1	1	1	1	1	0.02	1	1	1	1	1	1	0.88078	0.01886937
FAM104A	FALSE	FALSE	1	1	0.0316	1	1	1	1	1	1	1	1	1	1	1	1	1	1	1	1	1	1	1	1	1	1	1	1	1	1	0.23636853	0.04967684
ZMYM3	FALSE	FALSE	1	1	1	1	1	1	1	1	1	1	1	1	1	1	1	1	1	1	1	1	0.0239	1	1	1	1	1	1	1	1	0.92997795	0.01754962
CCND1	TRUE	TRUE	1	1	1	1	1	1	1	1	1	1	1	1	1	1	1	1	1	1	1	1	1	1	1	1	1	1	1	0.0189	1	0.75591551	0.02920761
HIST2H3D	FALSE	FALSE	1	1	1	1	1	1	1	0.9332	1	1	1	1	1	1	1	1	1	1	1	1	1	1	1	1	1	1	1	1	1	0.00856044	0.0569479
ARHGAP5	FALSE	FALSE	1	1	1	1	1	1	0.0454	1	1	1	1	1	0.5144	1	1	1	1	1	1	1	1	1	1	1	1	1	1	1	1	0.56724856	0.03986261
RARG	FALSE	FALSE	1	0.0964	1	1	1	1	1	0.0611	1	1	1	1	1	1	1	1	1	1	1	1	1	1	1	1	1	1	1	1	1	0.8627378	0.1211354
INPPL1	FALSE	FALSE	1	1	1	1	1	1	1	1	1	1	1	1	1	1	1	1	1	1	1	1	1	1	1	1	1	1	1	0.0348	1	0.46243413	0.0560946
RREB1	FALSE	FALSE	1	1	1	1	1	1	1	1	1	1	1	1	1	1	1	1	1	1	0.0532	1	1	1	1	1	1	1	1	1	1	0.64204259	0.03037634
SMC1A	FALSE	TRUE	1	1	1	1	1	1	1	1	1	1	1	0.0318	1	1	1	1	1	1	1	1	1	1	1	1	1	1	1	1	1	0.9674615	0.04217339
EFNB3	FALSE	FALSE	1	1	1	1	1	1	1	1	1	1	1	1	1	1	1	1	1	1	1	1	1	1	1	0.0829	1	1	1	1	1	0.15424995	0.11309836
OR11H12	FALSE	FALSE	1	1	1	1	1	1	1	1	1	1	1	0.5032	1	1	1	1	1	1	1	1	1	1	0.8679	1	1	1	1	1	1	0.02328304	0.14388396
DAZAP1	FALSE	FALSE	1	0.2586	1	1	1	1	1	1	1	1	1	1	1	0.4867	1	1	1	1	1	1	1	1	1	1	1	1	1	1	1	0.04677668	0.27298931
HIST2H2BE	FALSE	FALSE	1	1	1	1	1	1	1	1	1	1	1	1	1	1	1	1	1	1	1	1	1	1	1	1	1	1	1	1	1	0.01690256	0.10643592
MLH1	TRUE	FALSE	1	1	1	1	0.3027	1	1	1	1	1	1	1	1	1	1	1	1	1	1	1	1	1	1	0.5015	1	1	1	1	1	0.04939555	0.28742085
FOXP1	TRUE	FALSE	1	0.2134	1	1	1	1	1	1	1	1	1	1	1	1	1	1	1	1	1	1	0.8639	1	1	1	1	1	1	1	1	0.04614891	0.26562954
TAP2	FALSE	FALSE	1	1	1	1	0.0442	1	1	1	1	1	1	1	1	1	1	1	1	1	1	1	1	1	1	1	1	1	1	1	1	0.83859886	0.06379003
IL6ST	TRUE	FALSE	1	1	1	1	1	1	1	1	1	1	1	1	1	0.0572	1	1	1	1	1	1	1	1	1	1	1	1	1	1	1	0.82276275	0.055507
POM121L12	FALSE	FALSE	1	1	1	1	1	1	1	0.0497	1	1	1	1	1	1	1	1	1	1	1	1	1	1	1	1	1	1	1	1	1	0.64204259	0.09535185
C3orf70	FALSE	TRUE	1	0.0369	1	1	1	1	1	1	1	1	1	1	1	1	1	1	1	1	1	1	1	1	1	1	1	1	1	1	1	0.96571886	0.08933101
EZH2	TRUE	TRUE	1	1	1	1	1	1	1	1	1	1	1	0.0498	1	1	1	1	1	1	1	1	1	1	1	1	1	1	1	1	1	1	0.06550636
MAX	TRUE	FALSE	1	1	1	1	1	1	1	1	1	1	1	1	0.5591	1	0.2772	1	1	1	1	1	1	1	1	1	1	1	1	0.955	1	0.08022352	0.31979337
CDKN2C	TRUE	FALSE	1	1	1	1	1	1	1	1	1	1	1	1	0.0695	1	1	1	1	1	1	1	1	1	1	1	1	1	1	1	1	0.65751245	0.08442789
FAM58A	FALSE	FALSE	1	1	1	1	1	1	1	1	1	1	1	1	1	1	1	1	1	1	1	1	1	1	0.0694	1	1	1	1	1	1	0.77085709	0.0735539
PPP3CA	FALSE	FALSE	1	1	1	1	1	1	1	1	1	1	1	1	1	1	1	1	1	1	1	1	1	1	1	0.0583	1	1	1	1	1	0.94215312	0.07865408
PRRT2	FALSE	FALSE	1	1	1	1	0.0856	1	1	1	1	1	1	1	1	1	1	1	1	1	1	1	1	1	1	1	1	1	1	1	1	0.4533704	0.12061808
ERBB4	FALSE	FALSE	1	1	1	1	1	1	1	1	1	1	1	1	1	1	1	1	1	1	1	1	1	1	1	0.0843	1	1	1	1	1	0.52281716	0.11899019
MAP2K1	TRUE	TRUE	1	1	1	1	1	1	1	1	1	1	1	1	1	1	1	1	1	1	1	1	1	1	0.2819	1	1	1	1	1	1	0.06703449	0.30565172
SPEN	TRUE	TRUE	1	1	0.2662	1	1	1	1	1	1	1	1	1	1	1	1	1	1	1	1	1	1	1	1	1	1	1	1	1	1	0.0670257	0.37955474
NFE2L3	FALSE	FALSE	1	0.4837	0.8622	1	1	1	1	1	1	1	1	1	1	1	1	1	1	1	1	1	1	1	1	1	1	1	1	1	1	0.05594834	0.32178874
ACVR1B	FALSE	TRUE	1	1	1	1	1	1	1	1	1	1	1	1	1	1	1	1	1	1	1	1	1	1	1	0.8567	1	1	1	1	1	0.04618088	0.2682767
CUL4B	FALSE	TRUE	1	1	1	1	1	1	1	1	1	1	1	1	0.0998	1	1	1	1	1	1	1	1	1	1	1	1	1	1	1	1	0.98020349	0.12661588
CYP11B1	FALSE	FALSE	1	1	1	1	1	1	1	1	1	1	1	1	1	1	1	0.4218	1	1	1	1	1	1	1	1	1	1	1	1	1	0.07377262	0.40892246
HIST1H2AC	FALSE	FALSE	1	1	1	1	1	1	1	1	1	1	1	1	1	1	0.9144	1	1	1	1	1	1	1	1	1	1	1	1	1	1	0.05156905	0.30061579
MAZ	FALSE	FALSE	1	1	1	1	1	1	1	1	1	1	1	1	1	1	1	0.6776	1	1	1	1	1	1	1	1	1	1	1	1	1	0.06277202	0.35701781
GNG13	FALSE	FALSE	1	1	1	1	1	1	1	1	1	1	1	1	1	1	0.4492	1	1	1	1	1	1	1	1	1	1	1	1	1	1	0.07951057	0.4314204
POTEE	FALSE	FALSE	1	1	1	1	1	1	1	1	1	1	1	1	1	1	1	1	1	1	1	1	1	1	1	1	1	1	1	1	1	0.05476815	0.3148957
ZNF679	FALSE	FALSE	1	1	1	1	1	1	1	1	1	1	1	1	1	1	1	1	1	1	1	1	1	1	1	1	1	1	1	1	1	0.05709109	0.32897003
USP28	FALSE	FALSE	1	1	1	1	1	1	1	1	1	1	1	1	1	1	1	1	1	1	1	1	1	1	1	1	1	1	1	1	1	0.06137768	0.35008091
AMBRA1	FALSE	FALSE	1	1	1	1	1	1	1	1	1	1	1	1	1	1	1	1	1	1	1	1	1	1	1	1	1	1	1	1	1	0.06491668	0.36798701
MSH2	TRUE	FALSE	1	1	1	1	1	1	1	1	1	1	1	1	1	1	1	1	1	1	1	1	1	1	1	1	1	1	1	1	1	0.07216102	0.40268878
MYC	TRUE	FALSE	1	1	1	1	1	1	1	1	1	1	1	1	1	1	1	1	1	1	1	1	1	1	1	1	1	1	1	1	1	0.07642637	0.41816879
DYRK4	FALSE	FALSE	1	1	1	1	1	1	1	1	1	1	1	1	1	1	1	1	1	1	1	1	1	1	1	1	1	1	1	1	1	0.07642637	0.41831083
YLPM1	FALSE	FALSE	1	1	1	1	1	1	1	1	1	1	1	1	1	1	1	1	1	1	1	1	1	1	1	1	1	1	1	1	1	0.08781431	0.47293166
SMAD2	FALSE	TRUE	1	1	1	1	1	1	1	1	1	1	1	1	1	1	1	1	1	1	1	1	1	1	1	1	1	1	1	1	1	0.08968199	0.48516484

Table S3. [Related to Figure 4] List of 369 known cancer genes used in this study.

ABL1	*CCDC6*	*EIF1AX*	*HIST1H2BD*	*MED12*	*POLE*	*SMARCB1*	*UPF3A*
ACO1	*CCND1*	*EIF2S2*	*HIST1H3B*	*MED23*	*POT1*	*SMC1A*	*VHL*
ACVR1	*CD1D*	*ELF3*	*HIST1H4E*	*MEN1*	*POU2AF1*	*SMC3*	*WASF3*
ACVR1B	*CD58*	*EML4*	*HLA-A*	*MET*	*POU2F2*	*SMO*	*WT1*
ACVR2A	*CD70*	*EP300*	*HLA-B*	*MGA*	*PPM1D*	*SMTNL2*	*XIRP2*
ACVR2B	*CD79A*	*EPAS1*	*HLA-C*	*MLH1*	*PPP2R1A*	*SNX25*	*XPO1*
ADNP	*CD79B*	*EPHA2*	*HNF1A*	*MPL*	*PPP6C*	*SOCS1*	*ZBTB20*
AJUBA	*CDC27*	*EPS8*	*HOXB3*	*MPO*	*PRDM1*	*SOX17*	*ZBTB7B*
AKT1	*CDC73*	*ERBB2*	*HRAS*	*MSH2*	*PRKAR1A*	*SOX9*	*ZFHX3*
ALB	*CDH1*	*ERBB3*	*IDH1*	*MSH6*	*PSG4*	*SPEN*	*ZFP36L1*
ALK	*CDH10*	*ERCC2*	*IDH2*	*MTOR*	*PSIP1*	*SPOP*	*ZFP36L2*
ALPK2	*CDK12*	*ERG*	*IKBKB*	*MUC17*	*PTCH1*	*SPTAN1*	*ZFX*
AMER1	*CDK4*	*ESR1*	*IKZF1*	*MUC6*	*PTEN*	*SRC*	*ZMYM3*
APC	*CDKN1A*	*ETNK1*	*IL6ST*	*MXRA5*	*PTPN11*	*SRSF2*	*ZNF471*
APOL2	*CDKN1B*	*EZH2*	*IL7R*	*MYD88*	*PTPRB*	*STAG2*	*ZNF620*
ARHGAP35	*CDKN2A*	*FAM104A*	*ING1*	*MYOCD*	*QKI*	*STAT3*	*ZNF750*
ARHGAP5	*CDKN2C*	*FAM166A*	*INTS12*	*MYOD1*	*RAC1*	*STAT5B*	*ZNF800*
ARID1A	*CEBPA*	*FAM46C*	*IPO7*	*NBPF1*	*RACGAP1*	*STK11*	*ZNRF3*
ARID1B	*CHD4*	*FAT1*	*IRF4*	*NCOR1*	*RAD21*	*STK19*	*ZRSR2*
ARID2	*CHD8*	*FBXO11*	*ITGB7*	*NF1*	*RASA1*	*STX2*	
ARID5B	*CIB3*	*FBXW7*	*ITPKB*	*NF2*	*RB1*	*SUFU*	
ASXL1	*CIC*	*FGFR1*	*JAK1*	*NFE2L2*	*RBM10*	*TBC1D12*	
ATM	*CMTR2*	*FGFR2*	*JAK2*	*NIPBL*	*RET*	*TBL1XR1*	
ATP1A1	*CNBD1*	*FGFR3*	*JAK3*	*NOTCH1*	*RHEB*	*TBX3*	
ATP1B1	*CNOT3*	*FLG*	*KANSL1*	*NOTCH2*	*RHOA*	*TCEB1*	
ATP2B3	*COL2A1*	*FLT3*	*KCNJ5*	*NPM1*	*RHOB*	*TCF12*	
ATRX	*COL5A1*	*FOSL2*	*KDM5C*	*NRAS*	*RIT1*	*TCF7L2*	
AXIN1	*COL5A3*	*FOXA1*	*KDM6A*	*NSD1*	*RNF43*	*TCP11L2*	
AXIN2	*CREBBP*	*FOXA2*	*KDR*	*NT5C2*	*RPL10*	*TDRD10*	
AZGP1	*CRLF2*	*FOXL2*	*KEAP1*	*NTN4*	*RPL22*	*TERT*	
B2M	*CSDE1*	*FOXQ1*	*KEL*	*NTRK3*	*RPL5*	*TET2*	
BAP1	*CSF1R*	*FRMD7*	*KIT*	*NUP210L*	*RPS15*	*TG*	
BCLAF1	*CSF3R*	*FUBP1*	*KLF4*	*OMA1*	*RPS2*	*TGFBR2*	
BCOR	*CTCF*	*GAGE12J*	*KLF5*	*OR4A16*	*RPS6KA3*	*TGIF1*	
BHMT2	*CTNNA1*	*GATA1*	*KLHL8*	*OR4N2*	*RREB1*	*TIMM17A*	
BIRC3	*CTNNB1*	*GATA2*	*KMT2A*	*OR52N1*	*RUNX1*	*TNF*	
BMPR2	*CUL3*	*GATA3*	*KMT2B*	*OTUD7A*	*RXRA*	*TNFAIP3*	
BRAF	*CUL4B*	*GNA11*	*KMT2C*	*PAPD5*	*SELP*	*TNFRSF14*	
BRCA1	*CUX1*	*GNA13*	*KMT2D*	*PAX5*	*SETBP1*	*TOP2A*	
BRCA2	*CYLD*	*GNAQ*	*KRAS*	*PBRM1*	*SETD2*	*TP53*	
BRD7	*DAXX*	*GNAS*	*KRT5*	*PCBP1*	*SF3B1*	*TRAF3*	
C3orf70	*DDX3X*	*GNB1*	*LATS2*	*PDAP1*	*SGK1*	*TRAF7*	
CACNA1D	*DDX5*	*GNPTAB*	*LCTL*	*PDGFRA*	*SH2B3*	*TRIM23*	
CALR	*DIAPH1*	*GPS2*	*LZTR1*	*PDSS2*	*SLC1A3*	*TSC1*	
CARD11	*DICER1*	*GTF2I*	*MAP2K1*	*PDYN*	*SLC26A3*	*TSC2*	
CASP8	*DIS3*	*GUSB*	*MAP2K2*	*PHF6*	*SLC44A3*	*TSHR*	
CBFB	*DNM2*	*H3F3A*	*MAP2K4*	*PHOX2B*	*SLC4A5*	*TTLL9*	
CBL	*DNMT3A*	*H3F3B*	*MAP2K7*	*PIK3CA*	*SMAD2*	*TYRO3*	
CBLB	*EEF1A1*	*HIST1H1C*	*MAP3K1*	*PIK3R1*	*SMAD4*	*U2AF1*	
CCDC120	*EGFR*	*HIST1H1E*	*MAX*	*PLCG1*	*SMARCA4*	*UBR5*	
